# Methods for Assessing Therapeutic Innovation of Drugs: Changes at the Italian Medicine Agency

**DOI:** 10.1007/s43441-026-01021-y

**Published:** 2026-07-15

**Authors:** Domenico Motola, Nicola Montanaro, Massimiliano Broccoli, Victoria Nikitina

**Affiliations:** 1 Unit of Pharmacology, Department of Medical and Surgical Sciences, Alma Mater Studiorum University of Bologna, Bologna, Italy; 2 Former Professor of Pharmacology, Alma Mater Studiorum University Di Bologna, Bologna, Italy

**Keywords:** New drugs, Therapeutic innovation, Assessment methods, EMA, AIFA

## Abstract

**Introduction:**

Therapeutic drug innovation is a fundamental point for patients, healthcare professionals, and national health services. In Europe, the assessment of the therapeutic innovation of drug is establish to the individual national health agencies. In Italy, the Italian Medicines Agency (AIFA) was the first to adopt an algorithm for assessing the degree of therapeutic innovation of drugs in 2007. Afterwards, the AIFA has been issuing various versions of a method of assessment of therapeutic innovations, the most recent one being that issued in September 2025.

**Aim:**

To compare the results of the assessment of therapeutic innovation of drugs recently authorized by the European Medicines Agency (EMA), by using the former method (here called AIFA2007) vs the present one (here called AIFA2025), in order to determine which of the two was more selective.

**Results:**

38 new medicines authorized by the EMA between June 2023 and 2025 were evaluated. Among those analyzed with AIFA2007, only 3 achieved a degree of "important" therapeutic innovation (8%). Based on AIFA2025 criteria, as many as 12 drugs were classified as innovative (32%).

**Conclusion:**

The 2007 algorithm appeared to be much more selective in comparison to the recent one AIFA2025.

**Supplementary information:**

The online version contains supplementary material available at https://doi.org/10.1007/s43441-026-01021-y.

## Introduction

Therapeutic drug innovation is a fundamental point for patients, healthcare professionals, and national health services. The definition of therapeutic innovation, issued by ISDB in 2001 [1] is still up-to-date: a drug is considered therapeutically innovative when it offers additional clinical benefits compared with previously existing treatments. Of course, target disease and size of benefit are of paramount importance in assessing innovativeness of a new drug.

With the advent of advanced therapies (gene therapies, cell therapies, mRNA delivery systems, CRISPR-based gene therapy medicinal products, etc.), assessing the degree of drug innovation has become an even more demanding challenge. The degree of innovation of new drugs also has a direct economic impact on national health services, which face increasing difficulties in appropriate allocation of resources due to budget constraints. In Europe, the assessment of the therapeutic innovation of drug is established by the individual national health agencies. Since the early 2000s, numerous scientific methods have been proposed to evaluate and grade the therapeutic innovativeness of new drugs, each with advantages and disadvantages [2]. In Italy, the Italian Medicines Agency (AIFA) was the first to adopt an algorithm for assessing the degree of therapeutic innovation of drugs in 2007 [the link to the AIFA 2007 act is no longer available, so refer to the appendix 1 in the reference 3], adapting a method proposed by some of us (DM, NM) [3, 4]. Around the same time, the French and German agencies also developed their own methods for grading therapeutic innovation for the purposes of admitting new drugs to reimbursement [5]. Since 2008 onwards, following a drastic change of its general management [6, 7], the AIFA has been issuing various versions of a method of assessment of therapeutic innovations, with more or less important changes, resulting in the most recent proposal in September 2025. Presently, this last version is available only in Italian [8]), anyhow it follows the criteria described by Fortinguerra et al. in 2020 [9]. We have already expressed some concerns about that proposal [10]. The main features in this new method are (1) drugs considered for innovativeness are only those intended for diseases defined as having a medium/low epidemiological impact; (2) lack of a prearranged cascade of scrutiny of the various parameters used to assess the innovativeness; (3) outcome of evaluation as an all-or-none classification of a drug as innovative or not, unlike the previous methods, where innovativeness was ranked as modest, moderate, important; (4) drugs designated as innovative are recognized with significant economic and reimbursement benefit. This last point differs from what happens in France and Germany, where the evaluation of the degree of therapeutic innovation and the subsequent economic decision for reimbursement purposes are two distinct acts and carried out by two separate expert committees (for details, see https://www.g-ba.de/english/benefitassessment/ and https://www.has-sante.fr/jcms/c_2035651/en/methods-and-criteria-for-assessing-medicinal-products).

The aim of this study was to compare the results of the assessment of therapeutic innovation of a group of drugs authorized by the European Medicines Agency (EMA), by using the former method (here called AIFA2007) [11] vs the present one (here called AIFA2025) [8], in order to determine which of the two was more selective.

## Material and Methods

The drugs to be evaluated were retrieved from the European Medicines Agency's website by using the "Find medicine" function. Data on new human medicines with a positive opinion from June 1, 2023, to 30 June, 2025, consisting of orphan medicines, conditional approvals, exceptional circumstances, PRIME (priority medicines), and advanced therapy categories were downloaded. The freely available clinical information in the European Public Assessment Reports (EPARs) of the analyzed medicines was used to assign therapeutic innovation parameters (see Table S1 for details).

The AIFA2007 method consisted of an algorithm where the following three parameters, (1)–(3), were assigned in cascade, yielding to the overall score for innovation. Each parameter could take one of the following three levels, (a)–(c), in decreasing order of importance. (1) Disease seriousness: (a) drugs for serious diseases (a disease is serious if it meets one of the following criteria: it is fatal, it requires hospitalization, it is life-threatening or heavily disabling); (b) drugs for risk factors for serious diseases; (c) drugs for nonserious diseases. (2) Availability of treatments: (a) drugs for diseases without recognized standard treatment; (b) drugs for diseases where subsets of patients are less responsive to marketed drugs and/or other medical interventions; (c) drugs for diseases responsive to marketed drugs or other medical interventions; level (c) was further subdivided as follows: (c1) more effective or safer or with a better kinetics than existing drugs; (c2) mere pharmacological innovation, i.e. drugs with a new mechanism of action; (c3) mere technological innovation, i.e. a new chemical or biotechnological product with a therapeutic role similar to already existing ones). (3) Therapeutic effect: (a) major benefit on clinical end-points (e.g. increased survival rate and/or quality of life) or validated surrogate end points; (b) partial benefit on the disease (on clinical or validated surrogate end-points) or limited evidence of a major benefit (inconsistent results); (c) minor or temporary benefit on some aspects of the disease (e.g. only partial symptomatic relief of a serious disease) [3, 4].

The AIFA2025 method is available only in Italian [8]. Although this method is defined as new, it follows closely the method published by Fortinguerra et al. in 2020 [9]. According to these new criteria, only drugs intended for diseases with a medium or low epidemiological impact will be eligible for the evaluation of therapeutic innovation (however, without an explicit threshold for such epidemiological impact). The evaluation is based on three parameters: (1) Therapeutic need, (2) Added therapeutic value, and (3) Quality of clinical evidence. The first two parameters provide five possible categories in decreasing order of importance (maximum, important, moderate, poor, absent), while the final parameter, regarding the quality of evidence, provides four levels in decreasing order of relevance (high, moderate, low, very low). Refer to the mentioned publication by Fortinguerra et al. [9] for a more in-depth description of the various parameters. Based on the combination of the three parameters, the AIFA2025 method classify the evaluated drugs as innovative or not innovative. To be considered innovative, the scores for therapeutic need, added therapeutic value, and quality of evidence must be scored at least “moderate”. For orphan drugs, the quality of evidence may be “low”.

Two authors of this paper, DM and NM, independently assigned scores to each drug according to the one and the other method. In case of disagreement, the final scores were assigned through a consensus process (by discussion). All authors unanimously approved the final scores.

## Results

Thirty-eight new medicines were evaluated with a positive opinion by the EMA between June 2023 and June 2025. Table 1 lists the medicinal products with the name of the active substance, the trade name, the link to the EPAR, the therapeutic indication and the type of marketing authorization granted by the EMA (orphan medicine, PRIME, conditional approval, exceptional circumstances, advance therapy). Thirty of the 38 medicines were orphan medicines, 8 out of 38 were PRIME, 14 were authorized with conditional approval, five were authorized under exceptional circumstances, and 2 out of 38 were advanced therapies. In 15 of the 38 cases, the drugs had more than one type of marketing authorization, in one case four (orphan medicine, conditional approval, PRIME, advanced therapy).

**Table 1:** Medicinal products and the type of marketing authorization granted by the EMA (June 1, 2023, to 30 June, 2025)

Active substance	Medicinal product	Link EPAR	Therapeutic indication	Orphan Medicine	PRIME	Conditional approval	Exceptional circumstances	Advance therapy
Odevixibat	Kayfanda	Kayfanda (odevixibat)	Indicated for the treatment of cholestatic pruritus in Alagille syndrome (ALGS) in patients aged 6 months or older				✓	
Givinostat	Duvyzat	Duvyzat, INN-givinostat	Treatment of Duchenne muscular dystrophy (DMD) in ambulant patients, aged 6 years and older, and with concomitant corticosteroid treatment	✓		✓		
Vilobelimab	Gohibic	Gohibic, INN-vilobelimab	Indicated for treatment of adult patients with SARS-CoV-2-induced septic acute respiratory distress syndrome (ARDS) receiving invasive mechanical ventilation (IMV) or extracorporeal membrane oxygenation (ECMO)				✓	
Danicopan	Voydeya	Voydeya, INN-danicopan	Indicated as an add-on to ravulizumab or eculizumab for the treatment of signs or symptoms of extravascular haemolysis (EVH) in adult patients with paroxysmal nocturnal haemoglobinuria (PNH)	✓	✓			
Seladelpar	Lyvdelzi	Seladelpar Gilead, INN-seladelpar	Indicated for the treatment of primary biliary cholangitis (PBC) including pruritus in adults without cirrhosis or with compensated cirrhosis (Child–Pugh A) in combination with ursodeoxycholic acid (UDCA) who have an inadequate response to UDCA alone, or as monotherapy in those unable to tolerate UDCA	✓	✓	✓		
Zanidatamab	Ziihera	Ziihera. INN—zanidatamab	Indicated for the treatment of adults with previously treated, unresectable locally advanced or metastatic HER2-positive biliary tract cancer (BTC)	✓		✓		
Beremagene geperpavec	Vyjuvek	Vyjuvek, INN- beremagene geperpavec	Treatment of patients from birth with dystrophic epidermolysis bullosa (DEB) with mutation(s) in the collagen type VII alpha 1 chain (COL7A1) gene	✓	✓			✓
Palopegteriparatide	Yorvipath	Yorvipath, INN-palopegteriparatide	Parathyroid hormone (PTH) replacement therapy indicated for the treatment of adults with chronic hypoparathyroidism	✓				
Rezafungin	Rezzayo	Rezzayo Public Assessment Report	Treatment of invasive candidiasis in adults	✓				
Trametinib	Spexotras	Spexotras; INN-trametinib	In combination with dabrafenib is indicated for the treatment of paediatric patients aged 1 year and older with low-grade glioma (LGG) or High-grade glioma (HGG) with a BRAF V600E mutation who require systemic therapy	✓				
Dabrafenib	Finlee	Finlee; INN-dabrafenib	In combination with trametinib is indicated for the treatment of paediatric patients aged 1 year and older with low-grade glioma (LGG) or High-grade glioma (HGG) with a BRAF V600E mutation who require systemic therapy	✓				
Imetelstat	Rytelo	Rytelo, INN-imetelstat sodium;	Indicated as monotherapy for the treatment of adult patients with transfusion-dependent anaemia due to very low, low or intermediate risk myelodysplastic syndromes (MDS) without an isolated deletion 5q cytogenetic (non-del 5q) abnormality and who had an unsatisfactory response to or are ineligible for erythropoietin-based therapy	✓				
Momelotinib	Omjjara	Omjjara; INN-momelotinib	Indicated for the treatment of disease-related splenomegaly or symptoms in adult patients with moderate to severe anaemia who have primary myelofibrosis, post polycythaemia vera myelofibrosis or post essential thrombocythaemia myelofibrosis and who are Janus Kinase (JAK) inhibitor naïve or have been treated with ruxolitinib	✓				
Rozanolixizumab	Rystiggo	Rystiggo- INN-rozanolixizumab	Indicated as an add-on to standard therapy for the treatment of generalised myasthenia gravis (gMG) in adult patients who are anti-acetylcholine receptor (AChR) or anti-muscle-specific tyrosine kinase (MuSK) antibody positive	✓				
Elafibranor	Iqirvo	Iqirvo, INN-elafibranor	Indicated for the treatment of primary biliary cholangitis (PBC) in combination with ursodeoxycholic acid (UDCA) in adults with an inadequate response to UDCA, or as monotherapy in patients unable to tolerate UDCA	✓		✓		
Serplulimab	Hetronifly	Hetronifly, INN-serplulimab	In combination with carboplatin and etoposide is indicated for the first-line treatment of adult patients with extensive-stage small cell lung cancer (ES-SCLC)	✓				
Linvoseltamab	Lynozyfic	Lynozyfic- INN, linvoseltamab	Indicated as monotherapy for the treatment of adult patients with relapsed and refractory multiple myeloma who have received at least 3 prior therapies, including a proteasome inhibitor, an immunomodulatory agent, and an anti-CD38 monoclonal antibody, and have demonstrated disease progression on the last therapy			✓		
Sparsentan	Filspari	Filspari, INN-sparsentan	Indicated for the treatment of adults with primary immunoglobulin A nephropathy (IgAN) with a urine protein excretion ≥ 1.0 g/day (or urine protein-to-creatinine ratio ≥ 0.75 g/g	✓		✓		
Iptacopan	Fabhalta	Fabhalta, INN—iptacopan	Indicated as monotherapy in the treatment of adult patients with paroxysmal nocturnal haemoglobinuria (PNH) who have haemolytic anaemia	✓	✓			
Zolbetuximab	Vyloy	Vyloy, INN-zolbetuximab	In combination with fluoropyrimidine- and platinum-containing chemotherapy, is indicated for the first line treatment of adult patients with locally advanced unresectable or metastatic HER2 negative gastric or gastro-oesophageal junction (GEJ) adenocarcinoma whose tumours are Claudin (CLDN) 18.2 positive	✓				
Elranatamab	Elrexfio	ELREXFIO, INN-elranatamab	Indicated as monotherapy for the treatment of adult patients with relapsed and refractory multiple myeloma, who have received at least three prior therapies, including an immunomodulatory agent, a proteasome inhibitor, and an anti-CD38 antibody and have demonstrated disease progression on the last therapy		✓	✓		
Sotatercept	Winrevair	WINREVAIR, INN-sotatercept	In combination with other pulmonary arterial hypertension (PAH) therapies, is indicated for the treatment of PAH in adult patients with WHO Functional Class (FC) II to III, to improve exercise capacity	✓	✓			
Vamorolone	Agamree	Agamree, INN-vamorolone	Indicated for the treatment of Duchenne muscular dystrophy (DMD) in patients aged 4 years and older	✓				
Omaveloxolone	Skyclarys	Skyclarys, INN-omaveloxolone	The treatment of Friedreich’s ataxia in adults and adolescents aged 16 years and older	✓				
Belzutifan	Welireg	Welireg, INN—belzutifan	Is indicated as monotherapy for the treatment of adult patients with advanced clear cell renal cell carcinoma that progressed following two or more lines of therapy that included a PD-(L)1 inhibitor and at least two VEGF-targeted therapies			✓		
Repotrectinib	Augtyro	AUGTYRO, INN—repotrectinib	As monotherapy is indicated for the treatment of adult patients with ROS1-positive advanced non-small cell lung cancer (NSCLC).*			✓		
Efanesoctocog alfa	Altuvoct	H-5968-AR-en_revised	Treatment and prophylaxis of bleeding in patients with haemophilia A (congenital factor VIII deficiency). Altuvoct can be used for all age groups	✓				
Mirvetuximab soravtansine	Elahere	ELAHERE, INN-Mirvetuximab soravtansine	As monotherapy is indicated for the treatment of adult patients with folate receptor-alpha (FRα) positive, platinum-resistant high grade serous epithelial ovarian, fallopian tube, or primary peritoneal cancer who have received one to three prior systemic treatment regimens	✓				
Exagamglogene autotemcel	Casgevy	Casgevy; INN-exagamglogene autotemcel	Is indicated for the treatment of transfusion dependent β thalassemia (TDT) in patients 12 years of age and older for whom haematopoietic stem cell (HSC) transplantation is appropriate and a human leukocyte antigen (HLA) matched related HSC donor is not available.*	✓	✓	✓		✓
Polihexanide	Akantior	AKANTIOR—EPAR	Is indicated for the treatment of Acanthamoeba keratitis in adults and children from 12 years of age	✓				
Pegzilarginase	Loargys	Loargys, INN-pegzilarginase;	Is indicated for the treatment of arginase 1 deficiency (ARG1 D), also known as hyperargininemia, in adults, adolescents and children aged 2 years and older	✓			✓	
Odronextamab	Ordspono	Ordspono, INN-odronextamab	As monotherapy is indicated for the treatment of adult patients with relapsed or refractory follicular lymphoma (r/r FL) after two or more lines of systemic therapy.*	✓		✓		
Epcoritamab	Tepkinly	Tepkinly; INN-epcoritamab	As monotherapy is indicated for the treatment of adult patients with relapsed or refractory diffuse large B-cell lymphoma (DLBCL) after two or more lines of systemic therapy.*			✓		
rADAMTS13	Adzynma	Adzynma; INN-rADAMTS13	Treatment of congenital thrombotic thrombocytopenic purpura (cTTP) due to ADAMTS13 deficiency	✓			✓	
Tofersen	Qalsody	Qalsody, INN-tofersen	Is indicated for the treatment of adults with amyotrophic lateral sclerosis (ALS), associated with a mutation in the superoxide dismutase 1 (SOD1) gene	✓			✓	
Talquetamab	Talvey	Talvey; INN-talquetamab	Is indicated as monotherapy for the treatment of adult patients with relapsed and refractory multiple myeloma, who have received at least 3 prior therapies, including an immunomodulatory agent, a proteasome inhibitor, and an anti CD38 antibody and have demonstrated disease progression on the last therapy	✓	✓	✓		
Adagrasib	Krazati	Krazati, INN-adagrasib	As monotherapy is indicated for the treatment of adult patients with advanced non-small cell lung cancer (NSCLC) with KRAS G12C mutation and disease progression after at least one prior systemic therapy			✓		
Retifanlimab	Zynyz	Zynyz, INN-retifanlimab	Is indicated as monotherapy for the first-line treatment of adult patients with metastatic or recurrent locally advanced Merkel cell carcinoma (MCC) not amenable to curative surgery or radiation therapy	✓				

^*^Other therapeutic indications present in the SPC

Table 2 reports the individual scores of the criteria for therapeutic innovation based on the AIFA2007 and AIFA2025 criteria. Of the 38 drugs analyzed with AIFA2007, 3 achieved a degree of "important" therapeutic innovation (8%), 12 "moderate" (32%), 18 "modest" (47%), and 5 (13%) were classified as technological or pharmacological innovations. Based on AIFA2025 criteria, which divide drugs into innovative or non-innovative, 12 out of the 38 drugs analyzed were classified as innovative (32%); of these, 3 correspond to the level “important” of AIFA2007 while in the remaining 9 cases, the corresponding level of therapeutic innovation with AIFA2007 is “moderate” (8 drugs) and “modest” (1 drug). Table S1 reports all the notes supporting the individual scores assigned to all the drugs analyzed.

**Table 2:** Scores for the degree of therapeutic innovation according to AIFA 2007 vs 2025 criteria

			AIFA 2007^1^				AIFA 2025^2^			
Active substance	Medicinal product	Therapeutic indication	Disease seriousness	Availability of previous treatments	Therapeutic effect	Results	Therapeutic need	Added therapeutic value	Quality of clinical evidence	Results
Odevixibat	Kayfanda	Indicated for the treatment of cholestatic pruritus in Alagille syndrome (ALGS) in patients aged 6 months or older	A	B	C	Modest	Moderate	Poor	Low/very low	Innovation not assigned
Givinostat	Duvyzat	Treatment of Duchenne muscular dystrophy (DMD) in ambulant patients, aged 6 years and older, and with concomitant corticosteroid treatment	A	B	C	Modest	Important	Poor	Low/very low	Innovation not assigned
Vilobelimab	Gohibic	Indicated for treatment of adult patients with SARS-CoV-2-induced septic acute respiratory distress syndrome (ARDS) receiving invasive mechanical ventilation (IMV) or extracorporeal membrane oxygenation (ECMO)	A	B	C	Modest	Poor	Absent	Low/very low	Innovation not assigned
Danicopan	Voydeya	Indicated as an add-on to ravulizumab or eculizumab for the treatment of signs or symptoms of extravascular haemolysis (EVH) in adult patients with paroxysmal nocturnal haemoglobinuria (PNH)	A	B	C	Modest	Moderate	Moderate	Low	**Innovation assigned**
Seladelpar	Lyvdelzi	Indicated for the treatment of primary biliary cholangitis (PBC) including pruritus in adults without cirrhosis or with compensated cirrhosis (Child–Pugh A) in combination with ursodeoxycholic acid (UDCA) who have an inadequate response to UDCA alone, or as monotherapy in those unable to tolerate UDCA	A	B	B	Moderate	Moderate	Moderate	Moderate	**Innovation assigned**
Zanidatamab	Ziihera	Indicated for the treatment of adults with previously treated, unresectable locally advanced or metastatic HER2-positive biliary tract cancer (BTC)	A	B	C	Modest	Moderate	Poor	Low/very low	Innovation not assigned
Beremagene geperpavec	Vyjuvek	Treatment of patients from birth with dystrophic epidermolysis bullosa (DEB) with mutation(s) in the collagen type VII alpha 1 chain (COL7A1) gene	A	B	B	Moderate	Moderate	Moderate	Low	**Innovation assigned**
Palopegteriparatide	Yorvipath	Parathyroid hormone (PTH) replacement therapy indicated for the treatment of adults with chronic hypoparathyroidism	A	C3	A	Technological innovation	Moderate	Poor	Low/very low	Innovation not assigned
Rezafungin	Rezzayo	Treatment of invasive candidiasis in adults	A	C1	B	Modest	Poor	Absent	Low/very low	Innovation not assigned
Trametinib	Spexotras	In combination with dabrafenib is indicated for the treatment of paediatric patients aged 1 year and older with low-grade glioma (LGG) or High-grade glioma (HGG) with a BRAF V600E mutation who require systemic therapy	A	C1	A	Moderate	Important	Moderate	Low	**Innovation assigned**
Dabrafenib	Finlee	In combination with trametinib is indicated for the treatment of paediatric patients aged 1 year and older with low-grade glioma (LGG) or High-grade glioma (HGG) with a BRAF V600E mutation who require systemic therapy	A	C1	A	Moderate	Important	Moderate	Low	**Innovation assigned**
Imetelstat	Rytelo	Indicated as monotherapy for the treatment of adult patients with transfusion-dependent anaemia due to very low, low or intermediate risk myelodysplastic syndromes (MDS) without an isolated deletion 5q cytogenetic (non-del 5q) abnormality and who had an unsatisfactory response to or are ineligible for erythropoietin-based therapy	B	B	A	**Important**	Moderate	Moderate	Low	**Innovation assigned**
Momelotinib	Omjjara	Indicated for the treatment of disease-related splenomegaly or symptoms in adult patients with moderate to severe anaemia who have primary myelofibrosis, post polycythaemia vera myelofibrosis or post essential thrombocythaemia myelofibrosis and who are Janus Kinase (JAK) inhibitor naïve or have been treated with ruxolitinib	B	B	B	Moderate	Moderate	Moderate	Low	**Innovation assigned**
Rozanolixizumab	Rystiggo	Indicated as an add-on to standard therapy for the treatment of generalised myasthenia gravis (gMG) in adult patients who are anti-acetylcholine receptor (AChR) or anti-muscle-specific tyrosine kinase (MuSK) antibody positive	A	B	C	Modest	Moderate	Poor	Low/very low	Innovation not assigned
Elafibranor	Iqirvo	Indicated for the treatment of primary biliary cholangitis (PBC) in combination with ursodeoxycholic acid (UDCA) in adults with an inadequate response to UDCA, or as monotherapy in patients unable to tolerate UDCA	A	B	C	Modest	Poor	Moderate	Low/very low	Innovation not assigned
Serplulimab	Hetronifly	In combination with carboplatin and etoposide is indicated for the first-line treatment of adult patients with extensive-stage small cell lung cancer (ES-SCLC)	A	B	B	Moderate	Moderate	Important	Moderate	**Innovation assigned**
linvoseltamab	Lynozyfic	Indicated as monotherapy for the treatment of adult patients with relapsed and refractory multiple myeloma who have received at least 3 prior therapies, including a proteasome inhibitor, an immunomodulatory agent, and an anti-CD38 monoclonal antibody, and have demonstrated disease progression on the last therapy	A	B	C	Modest	Important	Poor	Low/very low	Innovation not assigned
Sparsentan	Filspari	Indicated for the treatment of adults with primary immunoglobulin A nephropathy (IgAN) with a urine protein excretion ≥ 1.0 g/day (or urine protein-to-creatinine ratio ≥ 0.75 g/g	A	B	B	Moderate	Moderate	Moderate	Low	**Innovation assigned**
Iptacopan	Fabhalta	Indicated as monotherapy in the treatment of adult patients with paroxysmal nocturnal haemoglobinuria (PNH) who have haemolytic anaemia	A	C1	B	Modest	Poor	Moderate	Moderate	Innovation not assigned
Zolbetuximab	Vyloy	In combination with fluoropyrimidine- and platinum-containing chemotherapy, is indicated for the first line treatment of adult patients with locally advanced unresectable or metastatic HER2 negative gastric or gastro-oesophageal junction (GEJ) adenocarcinoma whose tumours are Claudin (CLDN) 18.2 positive	A	B	B	Moderate	Moderate	Moderate	Low	**Innovation assigned**
Elranatamab	Elrexfio	Indicated as monotherapy for the treatment of adult patients with relapsed and refractory multiple myeloma, who have received at least three prior therapies, including an immunomodulatory agent, a proteasome inhibitor, and an anti-CD38 antibody and have demonstrated disease progression on the last therapy	A	B	C	Modest	Important	Poor	Low/very low	Innovation not assigned
Sotatercept	Winrevair	In combination with other pulmonary arterial hypertension (PAH) therapies, is indicated for the treatment of PAH in adult patients with WHO Functional Class (FC) II to III, to improve exercise capacity	A	C2	C	Pharmacological innovation	Moderate	Poor	Moderate	Innovation not assigned
Vamorolone	Agamree	Indicated for the treatment of Duchenne muscular dystrophy (DMD) in patients aged 4 years and older	A	C3	C	Technological innovation	Important	Poor	Moderate	Innovation not assigned
Omaveloxolone	Skyclarys	The treatment of Friedreich’s ataxia in adults and adolescents aged 16 years and older	A	A	C	Moderate	Important	Poor	Low/very low	Innovation not assigned
Belzutifan	Welireg	Welireg is indicated as monotherapy for the treatment of adult patients with advanced clear cell renal cell carcinoma that progressed following two or more lines of therapy that included a PD-(L)1 inhibitor and at least two VEGF-targeted therapies. *	A	B	B	Moderate	Moderate	Poor	Moderate	Innovation not assigned
Repotrectinib	Augtyro	Augtyro as monotherapy is indicated for the treatment of adult patients with ROS1-positive advanced non-small cell lung cancer (NSCLC). *	A	B	C	Modest	Moderate	Poor	Moderate	Innovation not assigned
Efanesoctocog alfa	Altuvoct	Treatment and prophylaxis of bleeding in patients with haemophilia A (congenital factor VIII deficiency). Altuvoct can be used for all age groups	A	C2	C	Pharmacological innovation	Minor	Important	Moderate	Innovation not assigned
Mirvetuximab soravtansine	Elahere	as monotherapy is indicated for the treatment of adult patients with folate receptor-alpha (FRα) positive, platinum-resistant high grade serous epithelial ovarian, fallopian tube, or primary peritoneal cancer who have received one to three prior systemic treatment regimens	A	B	A	**Important**	Moderate	Moderate	Moderate	**Innovation assigned**
Exagamglogene autotemcel	Casgevy	Casgevy is indicated for the treatment of transfusion dependent β thalassemia (TDT) in patients 12 years of age and older for whom haematopoietic stem cell (HSC) transplantation is appropriate and a human leukocyte antigen (HLA) matched related HSC donor is not available.*	A	B	C	Modest	Moderate	Poor	Low/very low	Innovation not assigned
Polihexanide	Akantior	is indicated for the treatment of Acanthamoeba keratitis in adults and children from 12 years of age	A	A	C	Moderate	**Important**	Absent	Low/very low	Innovation not assigned
Pegzilarginase	Loargys	is indicated for the treatment of arginase 1 deficiency (ARG1 D), also known as hyperargininemia, in adults, adolescents and children aged 2 years and older	A	A	B	**Important**	Moderate	Moderate	Low/very low	**Innovation assigned**
Odronextamab	Ordspono	as monotherapy is indicated for the treatment of adult patients with relapsed or refractory follicular lymphoma (r/r FL) after two or more lines of systemic therapy.*	A	B	C	Modest	Moderate	Poor	Low/very low	Innovation not assigned
Epcoritamab	Tepkinly	as monotherapy is indicated for the treatment of adult patients with relapsed or refractory diffuse large B-cell lymphoma (DLBCL) after two or more lines of systemic therapy.*	A	B	C	Modest	Poor	Poor	Moderate	Innovation not assigned
rADAMTS13	Adzynma	Treatment of congenital thrombotic thrombocytopenic purpura (cTTP) due to ADAMTS13 deficiency	A	B	C	Modest	Moderate	Absent	Low/very low	Innovation not assigned
Tofersen	Qalsody	is indicated for the treatment of adults with amyotrophic lateral sclerosis (ALS), associated with a mutation in the superoxide dismutase 1 (SOD1) gene	A	B	C	Modest	Maximum	Poor	Low/very low	Innovation not assigned
Talquetamab	Talvey	is indicated as monotherapy for the treatment of adult patients with relapsed and refractory multiple myeloma, who have received at least 3 prior therapies, including an immunomodulatory agent, a proteasome inhibitor, and an anti CD38 antibody and have demonstrated disease progression on the last therapy	A	B	C	Modest	Important	Poor	Low/very low	Innovation not assigned
Adagrasib	Krazati	as monotherapy is indicated for the treatment of adult patients with advanced non-small cell lung cancer (NSCLC) with KRAS G12C mutation and disease progression after at least one prior systemic therapy	A	B	C	Modest	Moderate	Poor	Low/very low	Innovation not assigned
Retifanlimab	Zynyz	is indicated as monotherapy for the first-line treatment of adult patients with metastatic or recurrent locally advanced Merkel cell carcinoma (MCC) not amenable to curative surgery or radiation therapy	A	C2	C	Pharmacological innovation	Moderate	Poor	Low/very low	Innovation not assigned

**1** AIFA 2007: Seriouness of the disease: therapeutic agents for (A) serious diseases (a disease is serious if it meets one of the following criteria: it is fatal, it requires hospitalization, it is life-threatening or heavily disabling; (B) risk factors for serious diseases; (C) non-serious diseases. The scores for availability of previous treatments were: A = drugs for diseases without recognized standard treatment at the time of their approval; B = drugs for diseases where subsets of patients are less responsive to marketed drugs and/or other medical interventions, and C = drugs for responsive diseases to marketed drugs or other medical interventions. Class C drugs were further divided into C 1 (more effective or safer than existing drugs), C 2 (mere pharmacological innovation, i.e. drugs with better kinetics or new mechanism of action), and C 3 (mere technological innovation, i.e. a new chemical or biotechnological product with similar therapeutic role to already existing ones). The therapeutic effect scores were: 'A' = major benefit on clinical end-points (e.g. increased survival rate and/or quality of life) or validated surrogate end-points; 'B' = partial benefit on the disease (on clinical or validated surrogate end-points) or limited evidence of a major benefit (inconsistent results); and 'C' = minor or temporary benefit on some aspects of the disease. Overall scores for therapeutic innovation (‘A’, important; 'B', moderate; ‘C’, modest) were assigned according to the algorithm

**2** AIFA 2025: The evaluation is based on three parameters: (1) Therapeutic need, (2) Added therapeutic value, and (3) Quality of clinical evidence. The first two parameters provide five possible categories in decreasing order of importance (Maximum, Important, Moderate, Poor, Absent), while the final parameter, regarding the quality of evidence, provides four levels in decreasing order of relevance (high, moderate, low, very low). Please refer to the publication by Fortinguerra et al. for a more in-depth discussion of the various parameters. Based on the combination of the three parameters, the AIFA 2025 method classify the evaluated drugs as innovative or not innovative. To be considered innovative, the scores for therapeutic need, added therapeutic value, and quality of evidence must be at least MODERATE. For orphan drugs, the quality of evidence can also be LOW

^*^Other therapeutic indications present in the SPC

## Discussion

Evaluating the therapeutic innovation of new and increasingly expensive drugs (i.e., assessing whether they offer additional benefits clinically significant) is a crucial aspect of any public healthcare system. Such technical and scientific assessments also determine the allocation of economic resources, which are increasingly lacking in relation to the population's therapeutic needs. There is no a single method for assessing the degree of therapeutic innovation of a given drug, although the few national regulatory agencies that perform this assessment for internal regulatory purposes (France, Germany, Italy) [5] use the same criteria (seriousness of the disease, availability of other treatments, extent of additional clinical benefit), albeit reconfigured in different ways.

Since 2007, AIFA has changed the system for evaluating these requirements several times, ultimately revising the same criteria but always defining them as new. The first and the latest of these methods (AIFA2007 and AIFA2025), however, show marked differences, which lies primarily in terms of grading of therapeutic innovation: in the AIFA2007 algorithm, innovation is graded from important to modest, down to pharmacological and technological innovation (i.e., in the latter two cases, no therapeutic innovation), while the AIFA2025 approach only leads to a yes/no innovation, with regard to the overall assessment. It is true, however, that for both approaches, the intermediate steps are graded. Regarding admission to reimbursement, this seems clearer in the AIFA2025 approach, since the overall evaluation is a yes/no, while in the AIFA2007 approach a different group of experts subsequently decided whether only "important" innovation would have been reimbursed. Another difference between the two approaches is the presence or absence of a defined sequence. The AIFA2025 approach does not clearly establish how the two main criteria (therapeutic need and added therapeutic value), with their five levels (maximum, important, moderate, poor, and absent), interact to achieve the qualification of an innovative drug, while the AIFA2007 method presented a precise path to achieve a graduated level of innovativeness. Thus, the 2007 algorithm appears to be much more selective; in fact, only 3 out of the 38 drugs analyzed achieved major therapeutic innovation status, unlike the 2025 method, where 12 out of 38 were classified as innovative. This aspect has general consequences since an innovation evaluation method plays a crucial role in balancing timely access with the sustainability of healthcare resources. A less selective assessment framework, as the 2025 AIFA approach may potentially represent, could increase the likelihood of classifying medicines with still immature clinical evidence as innovative. Such an approach may have important implications for healthcare systems, particularly in terms of resource allocation and opportunity costs, as financial resources dedicated to highly uncertain therapies may reduce the availability of funding for interventions supported by more robust evidence. Furthermore, there could also be a tendency for pharmaceutical companies to be less scrupulous when applying in submitting for recognition of therapeutic innovation of their medicines.

A limitation of this study is that the evaluation of a new drug's innovativeness is generally carried out collectively by a scientific committee. On the contrary, in this paper, the assessment was performed solely by two of the authors (NM and DM), and this may have led to results perhaps different from those that a regulatory body could produce. However, the present one was no more than a scientific exercise, performed for the purpose of comparing the two methods of innovativeness assessment, and certainly not with the claim of taking the place of a regulatory agency. This paper also provided an opportunity to evaluate the degree of innovation of drugs recently approved by the EMA (from June 1, 2023, to June 30, 2025) belonging to the categories of orphan medicine, conditional approval, exceptional circumstances, PRIME (priority medicines), and advanced therapy. Despite the different evaluation severity of the two approaches, most recently approved medicines do not reach anyway the level of full innovation.

Some comparisons with official evaluations can also be made. In fact, the 2024 AIFA evaluation is available for the drug Casgevy(R) (exagamglogene autotemcel, indicated for the treatment of transfusion-dependent β-thalassemia) [12]. In the present analysis, the drug received by us a "modest" rating according to our use of the 2007 criteria judging “minor” the added therapeutic value. On the contrary, in the AIFA's official evaluation, the drug was classified innovative: this because the added therapeutic value was considered “important criterion differs significantly: we assigned it a "minor" rating while the official evaluation was "important," although in the official explanatory notes AIFA expressed some major reservations on the therapeutic value of the drug.

Another comparison of our assessment and that officially issued by AIFA concerns the drug givinostat (DUVYZAT®) [13], authorized for the treatment of Duchenne muscular dystrophy (DMD) in patients 6 years of age and older who are able to walk and are receiving concomitant corticosteroid treatment. In our evaluation with the use of the 2007 criteria we classified the innovation of the drug as “modest” and with the use of 2025AIFA criteria, the innovation was “not assigned". On the contrary, the official AIFA assessment assigned to the drug the status of “innovative”, even if—also in this case- the official note accompanying the innovative classification of the drug expressed some major reservations of the therapeutic role of the drug.

Such indulgent official assignments of the status of “nnovative” according to the new 2025 criteria are not without consequences, since, drugs classified as innovative directly obtain additional commercial and economic benefits, i.e. (1) access to the resources of a special fund for innovative drugs, (2) suspension of legal reductions in drug prices, (3) immediate inclusion in the Therapeutic Formularies of the Italian regions, (4) exclusion from the calculation of the overall amount of pharmaceutical expenditure for pay-back procedures [14]. Furthermore, it should not be forgotten that the European Union already offers a range of incentives to encourage the development of orphan medicines [15], such as protocol assistance, market exclusivity once the medicine is on the market, fee reductions, and research funding. It has historically been argued that, without incentives, pharmaceutical companies would be unlikely to invest due to the limited economic return. However, it is difficult to sustain today, as stated for example by Sophie Cousins [16], for the drug Trikafta® (an innovative and highly effective combination therapy for cystic fibrosis), Vertex Pharmaceuticals, which holds a monopoly on this class of medicines, recorded more than 11 billion dollars in product revenue in 2024, largely driven by the Trikafta (Kaftrio® in Europe) sale [16]. We could therefore say, "Orphans, yes, but rich."

Unlike the former procedures in the previous years, where a committee of experts judged regarding the innovation and a separate body treated the conditions of reimbursement, now, with the the new method AIFA2025 we have to do with a hybrid scientific and economic procedure, where, as it is stated, only drugs targeting diseases with medium-to-low prevalence are eligible for the evaluation process, thus excluding drugs for widespread diseases (e.g., diabetes, hypertension, COPD) from the innovativeness classification, even in the hypothetical case of drugs that could represent a significant therapeutic breakthrough in these diseases. Such option does not appear explicitly justified in the official AIFA document [8]. Anti-infective drugs for infections caused by multidrug-resistant germs are also placed in a separate category, for which the innovativeness requirement is not assessed. For example, lenacapavir is a game changer in the treatment and prevention of HIV. In the PURPOSE 1 and 2 clinical trials, only two subcutaneous injections per year of lenacapavir prevented HIV infection by 96–100% in various populations across multiple countries [17]. Compared to previously available options, lenacapavir is clearly an innovative drug, also in terms of frequency of administration and therefore patient compliance.

It should also be considered that the focus on orphan drugs resulting from the 2025 criteria may also conceal pitfalls. The development of these drugs is clearly more critical than others due to the small sample sizes of pre-registration clinical trials, the lack of comparators, the use of historical controls, etc. In some cases, despite extensive clinical experience, these drugs have failed to meet the expectations of patients and physicians. For example, ataluren (Translarna®) was authorized by the EMA in 2014 for the treatment of patients with Duchenne muscular dystrophy. At the time of ataluren's approval, its efficacy was highly uncertain, and the EMA's CHMP initially did not authorized the drug. However, the CHMP revised its opinion, and in May 2014 proposed a conditional marketing authorization, requiring the company to conduct new studies to confirm the drug's benefits. Ten years later, in October 2024, following a review of the available data, the CHMP confirmed the recommendation not to renew the marketing authorization for ataluren, as the assessments conducted concluded that the drug's efficacy was not confirmed. Therefore, a drug of dubious efficacy, also with some adverse effects, and characterized by a very high cost, was used and reimbursed for a decade [18]. Another example is the drug voxelotor (Oxbryta®), indicated for the treatment of hemolytic anemia due to sickle cell disease. The drug was approved by the EMA in 2021. Voxelotor obtained authorization based on a new surrogate outcome: an increase in hemoglobin of at least 1.0 g/dL at week 24 compared to baseline. In 2024, Pfizer announced the voluntary withdrawal of voxelotor from the market, due to an imbalance between vaso-occlusive crises and fatal events, indicating that the drug's benefits no longer outweighed the risks, without providing further information [18].

Currently, three European regulatory authorities (France, Germany, and Italy) also perform this evaluation for pricing and reimbursement purposes. There are several recent comparisons available in the literature in this regard [5, 19, 20]. In France and Germany, the committees responsible for the technical-scientific assessment of the degree of innovation (using methods very similar to the AIFA2007 criteria) are separate from those who decide on prices and progressive reimbursement levels proportionate to the degree of innovation. All drugs authorized by the EMA or national authorities are eligible for these evaluations. In Italy, the technical-scientific assessment and the allocation of price and reimbursement are now assigned to a single committee, with limitations on the type of drug that can be evaluated and, where applicable, access the various economic and commercial benefits provided. Furthermore, given the restriction on the evaluation of drugs intended for medium–low impact diseases, the use of GRADE also appears very critical. The use of GRADE for the quality of evidence is absolutely legitimate but appears increasingly difficult to apply given the rapid progress of pharmacological therapies, particularly orphan drugs but above all for the already existing personalized therapies with the increasingly frequent use of so-called N-of-1 or N-of-few trials [21], etc. In this regard, it's worth briefly focusing on some very interesting and recent European regulatory and scientific initiatives: for rare diseases, there is the REALISE-D initiative (a consortium of universities, industry, and national and supranational regulatory agencies, https://realised-ihi.eu/successful-kick-off-meeting-for-the-realised-project-in-vienna/) aimed at transforming/updating/improving clinical trials for rare and ultra-rare diseases to accelerate the development of new treatments and improve outcomes for over 30 million people with rare diseases in Europe. The first meeting (April 2025) also laid the foundation for the pioneering work that will take place throughout the project to define new standards for clinical trials for rare and ultra-rare diseases, through a partnership of nearly 40 public and private organizations. Furthermore, orphan diseases, due to their rare nature, do not occur evenly among European countries, but their regional prevalence varies significantly. Thus, national uptake and HTA work are necessary to confirm the need and a reasonable price tag in the specific area. A second joint initiative between the EMA, national regulatory agencies (Spain, Germany, France, and others), and HTA agencies (Spain, Norway, Germany, and others), entitled "Joint positions for better evidence in approval and benefit assessments," published a position paper (https://www.iqwig.de/en/presse/iqwig-comments/1-april-2025.html) that affirms the need for drug manufacturers to conduct clinical trials aimed at both regulatory approval and effectiveness assessments (see also the website European HTA agencies and the EMA developed joint positions for better evidence).

## Conclusion

In conclusion, the comparison between the AIFA2007 and AIFA2025 methods highlights two profoundly different approaches to the assessment of therapeutic innovation. While the 2007 model adopted a more selective and graded scientific evaluation, the 2025 system appears broader and more directly connected to reimbursement and economic incentives. This shift may facilitate faster access to innovative therapies, particularly for rare diseases, but it also risks granting innovation status to medicines supported by immature or uncertain clinical evidence. The experience with several orphan and conditionally approved drugs demonstrates how early enthusiasm may later be contradicted by limited long-term efficacy or safety data. In this context, the balance between rapid access, scientific rigor, and sustainability of healthcare resources becomes increasingly critical. Moreover, restricting innovativeness assessments mainly to low-prevalence diseases introduces important ethical and policy questions, especially when highly transformative therapies for common diseases may remain excluded from the same benefits. Future evaluation systems should therefore aim for greater transparency, methodological consistency, and integration between regulatory approval and health technology assessment. A broader European coordination in therapeutic innovation assessment could also help ensure equitable access to effective treatments while preserving the sustainability of national healthcare systems.

## Supplementary Information

Below is the link to the electronic supplementary material.

Supplementary file1 (DOCX 89 kb)

## Data Availability

The data that support the findings of this study are available from the corresponding author upon reasonable request. The manuscript does not contain clinical studies or patient data. For this type of study, ethics committee approval and formal consent are not required.
