# Supplementary material for: Methods for Assessing Therapeutic Innovation of Drugs: Changes at the Italian Medicine Agency
Source: Ther Innov Regul Sci. 2026 Jul 15;60(6):1759–84. doi: 10.1007/s43441-026-01021-y (PMC13722619; doi:10.1007/s43441-026-01021-y)
Supplement: Supplementary file 1 [file 43441_2026_1021_MOESM1_ESM.docx]

**Table S1: scores for therapeutic innovation according to AIFA2007 and AIFA2025 methods; and in brackets the notes supporting the individual scores.**

The notes in brackets are based on the information in the EPAR

| **Active substance** | **Medicinal product** | **Link EPAR** | **Type of authorisation** | **Therapeutic indication** | **Disease Seriousness** | **Availability of previous treatments** | **Therapeutic effect** | **Result** | **Therapeutic need** | **Added therapeutic value** | **Quality of clinicl evidence** | **Result** |
| --- | --- | --- | --- | --- | --- | --- | --- | --- | --- | --- | --- | --- |
| Odevixibat | Kayfanda | [Kayfanda (odevixibat)](https://www.ema.europa.eu/en/documents/assessment-report/kayfanda-epar-public-assessment-report_en.pdf) | Exceptional circumstances | Indicated for the treatment of cholestatic pruritus in Alagille syndrome (ALGS) in patients aged 6 months or older. | A (symptom that compromises the quality of life) | B (There are previous treatments of limited effectiveness) | C (modest) | **A - B - C = modest** | Moderate (for the same disease, from two months of age there is another treatment, maralixibat, with surrogate benefits, namely a reduction in the level of bile acids in the blood.) | Minor (24-week clinical study in 45 patients vs. placebo, the efficacy of odevixibat in ALGS patients is based on the results of one pivotal, double-blinded, randomised, placebo-controlled phase III study and a long-term extension study. The study population includes male and female patients with ALGS who have pruritus and elevated baseline bile acid levels. The primary endpoint is the Change from baseline in scratching to Month 6 (Weeks 21 to 24) as measured by the Albireo ObsRO caregiver instrument, an observer/caregiver reported outcome to measure pruritus.A significant difference in a decrease from baseline to week 24 in scratching score was observed for the odevixibat (-1.66) group versus the placebo group (-0.79), with an LS mean difference -0.88, 95% CI -1.44, -0.33, one-sided p-value 0.0012. Taken together, the presented data support clinically relevant but modest treatment effects on pruritus and associated sleep problems. The pharmacodynamic effect of odevixibat is supported by the sustained decrease in serum bile acids. No clear effects were observed on dyslipidaemia, xanthomas, and growth. More importantly, there was no indication of improvements in liver function markers, such as ALT/AST and bilirubin.) | Very low | **Innovation not assigned** |
| Givinostat | Duvyzat | [Duvyzat, INN-givinostat](https://www.ema.europa.eu/en/documents/assessment-report/duvyzat-epar-public-assessment-report_en.pdf) | Conditional approval, Orphan drug | Treatment of Duchenne muscular dystrophy (DMD) in ambulant patients, aged 6 years and older, and with concomitant corticosteroid treatment. | A (serious, potentially life-threatening) | B (none, the drug is in addition to corticosteroids) | C (modest) | **A - B - C = modest** | Important (No medical cure exists for DMD, and the disease has a poor prognosis. Treatment is still centered on corticosteroid therapy, e.g. glucocorticoids, prevention of contractures, and medical care of  cardiomyopathy and respiratory compromise. Recent studies confirm the benefits of starting glucocorticoids in younger children, before significant  physical decline) | Minor (For the main evidence of efficacy, a single phase 3, multicentre, randomised, double-blind, parallel group, placebo (overall n = 61; target population n = 39) controlled study of 18 months treatment duration to assess the efficacy and safety of givinostat, in ambulant boys with Duchenne muscular dystrophy (DMD) has been submitted. The primary endpoint was the mean change in time to climb 4 stairs (4SC) before and after 18 months of treatment of givinostat versus placebo. While the time to 4SC increased in the givinostat group from 3.39 (SD 1.162) to 4.70 (SD 3.154) seconds it increased even more in the placebo group from 3.48 (SD 11.158) to 6.37 (SD 7.698) seconds. The evidence for efficacy was generated in one pivotal study. Post-hoc analyses for the primary, key secondary and velocity endpoints to address uncertainties with respect to the primary analysis using more conservative missing data handling strategies failed to show statistical significance) | Very low | **Innovation not assigned** |
| Vilobelimab | Gohibic | [Gohibic, INN-vilobelimab](https://www.ema.europa.eu/en/documents/assessment-report/gohibic-epar-public-assessment-report_en.pdf) | Exceptional circumstances | Indicated for treatment of adult patients with SARS-CoV-2-induced septic acute respiratory distress syndrome (ARDS) receiving invasive mechanical ventilation (IMV) or extracorporeal membrane oxygenation (ECMO). | A | B (for patients who do not benefit from available treatments) | C (modest) | **A - B - C = modest** | Minor (WHO recommends the use of systemic corticosteroids for severe or critical COVID-19 disease. Moreover, interleukin-6 (IL-6) receptor blockers such as tocilizumab, are recommended given in combination with corticosteroids. It is also recommended to use venous thromboembolism prophylaxis in hospitalised patients who require mechanical ventilation or ECMO (NIH COVID-19 Treatment Guidelines). The mortality of patients with COVID-19 under MV/ECMO remains high and therapeutic options are inadequate. New therapeutic principles to prevent disease progression and reduce mortality in late-stage COVID-19 pneumonia could therefore be useful (European Respiratory Society). | Absent (Phase III was a 1:1 randomised, double-blind, placebo-controlled multicentre study evaluating vilobelimab in adult patients with Sars-Cov-2 who were mechanically ventilated, and had been so for less than 48 hours at the time of randomisation. Moreover, patients had a PaO2 / FiO2 ratio of < 200 and > 60 at randomisation. There were 178 patients in the vilobelimab group and 191 in the placebo group. Patients were treated with a maximum of 6 intravenous (IV) doses of VILO 800 mg + SOC (Arm A) or Placebo + SOC (Arm B) at Days 1, 2, 4, 8, 15, and 22, as long as the patient was still hospitalised. The primary endpoint was 28-day all-cause mortality. Importantly, the pre-specified primary analysis of 28-day all-cause mortality, which was a sitestratified Cox regression model and adjusted for age, did not reach statistical significance. The proportion of patients with 28-day mortality was 31.65% vs. 41.59% in the vilobelimab and placebo groups, respectively (HR 0.728 [95% CI: 0.502, 1.056], p-value =0.0941). Thus, this pivotal study did not meet the primary endpoint and is formally not a positive study. The PANAMO study is formally not a statistically positive study. Thus, the statistical strength of evidence is lower than what is normally anticipated.) | Very low | **Innovation not assigned** |
| Danicopan | Voydeya | [Voydeya, INN-danicopan](https://www.ema.europa.eu/en/documents/assessment-report/voydeya-epar-public-assessment-report_en.pdf) | Orphan drug, Prime - priority medicine | Indicated as an add-on to ravulizumab or eculizumab for the treatment of signs or symptoms of extravascular haemolysis (EVH) in adult patients with paroxysmal nocturnal haemoglobinuria (PNH). | A | B | C (modest, surrogate endpoints, hemoglobinemia) | **A - B - C = modest** | Moderate (The current standard of care for PNH is treatment with eculizumab or ravulizumab; both monoclonal antibodies that are designed to target C5 complement protein. Both are approved to treat adults and children with paroxysmal nocturnal haemoglobinuria in EU since 2007 and 2019 respectively. While IVH is controlled by C5 inhibition (as indicated by LDH < 1.5 × ULN), a small subset of patients with PNH who achieve durable IVH control and associated disease control with ravulizumab or eculizumab may experience emergence of clinically significant EVH. | Moderato (The main evidence in support of this application is the Phase 3 study ALXN2040-PNH-301. The study ALXN2040-PNH-301 is a randomised, double-blind, placebo-controlled Phase 3 study in patients with PNH and clinically significant evidence of extravascular haemolysis while on treatment with a C5 inhibitor. The primary endpoint of the study was change in Hgb from baseline to week 12. Transfusion avoidance (TA) is considered a relevant objective of treatment and, in fact, it was suggested to be included as a co-primary endpoint, though this recommendation was not followed. All the efficacy analyses initially provided were based on the interim efficacy analysis set which is comprised by 63 patients (42 danicopan arm and 21 placebo arm). The LS mean (SE) change from baseline in Hgb at week 12 using MMRM was 29.40 (2.107) g/L in the danicopan group compared with 4.96 (3.128) g/L in the placebo group. The LS mean difference (SE) between the two groups was 2.44 g/dL; p<0.0001. The re-andomisation test analysis showed statistically significant results (p = 0.0007). An increase of 2 g/dL (20 g/L) can be considered of clinical relevance. The results in the key secondary endpoints supported the primary analysis). | Low | **Innovation assigned** |
| Seladelpar | Lyvdelzi | [Seladelpar Gilead, INN-seladelpar](https://www.ema.europa.eu/en/documents/assessment-report/seladelpar-gilead-epar-public-assessment-report_en.pdf) | Conditional approval, Orphan drug, PRIME - Priority medicine | Indicated for the treatment of primary biliary cholangitis (PBC) including pruritus in adults without cirrhosis or with compensated cirrhosis (Child-Pugh A) in combination with ursodeoxycholic acid (UDCA) who have an inadequate response to UDCA alone, or as monotherapy in those unable to tolerate UDCA | A | B (Drug for patients unresponsive to conventional treatment) | B (moderate, as assessed on surrogate endpoint, pruritus) | **A - B - B = moderate** | Moderate (The first-line therapy for PBC is UDCA, a noncytotoxic bile acid that has been the mainstay of treatment for more than 20 years. However, despite receiving UDCA, up to 40% of patients have persistent elevation of ALP and/or bilirubin and are considered inadequate responders (Corpechot 2008). Intolerance to UDCA may also occur and is seen in up to 5% of patients. | Moderate (Pivotal Phase 3 study RESPONSE was a phase 3, double-blind, randomised, placebo-controlled, multicentre study conducted in subjects with a diagnosis of PBC and an inadequate response or intolerance to UDCA. The study  evaluated oral seladelpar 10 mg once daily versus placebo over 52 weeks. The primary endpoint was a composite of cholestatic biomarkers (ALP and bilirubin). In the pivotal study, a total of 360 subjects were screened and 193 subjects were randomised  (seladelpar n=128, placebo n=65) and all received their intended study treatment. 79/128 (61.7%) in the seladelpar group and 13/65 (20%) in the placebo group achieved the composite biochemical primary endpoint (p<0.0001). The first key secondary endpoint, normalisation of ALP, was reached in 32/128 (25%) of the seladelpar treated patients and in none (0%) of the placebo treated patients. The applicant demonstrated a statistically significant improvement in pruritus for the treatment of PBC  with Seladelpar Gilead) | Moderate | **Innovation assigned** |
| Zanidatamab | Ziihera | [Ziihera. INN - zanidatamab](https://www.ema.europa.eu/en/documents/assessment-report/ziihera-epar-public-assessment-report_en.pdf) | Conditional approval, Orphan drug | Indicated for the treatment of adults with previously treated, unresectable locally advanced or metastatic HER2-positive biliary tract cancer (BTC) | A | B (Second-line drug for patients who cannot be operated on and who have not responded to previous conventional treatment) | C (Preliminary results to be confirmed: clinically relevant overall response rate of 51.6%) | **A - B - C = modest** | Moderate (There are no approved agents for HER2-amplified or HER2-expressing BTC. The current treatment for these  patients after a first-line gemcitabine-containing regimen is cytotoxic chemotherapy, which does not provide  a satisfactory disease prognosis) | Minor (The efficacy results of the pivotal single arm study 203 of zanidatamab monotherapy for the treatment of advanced HER2-positive ICH 3+ BTC showed a clinically relevant overall response rate of 51.6% (95%CI: 38.6; 64.5), which appears durable with a median DoR of 14.92 months (95%CI: 7.39; 24.0). The CHMP considers the following measures necessary to address the missing efficacy data in the context of a conditional MA: In order to confirm the efficacy and safety of zanidatamab in the treatment of adults with unresectable locally advanced or metastatic HER2-positive biliary tract cancer previously treated with at least one prior line of systemic therapy, the MAH should submit the results of the ongoing open-label phase III randomised clinical study JZP598-302 to evaluate the efficacy and safety of zanidatamab plus standard-of-care therapy versus standard-of-care therapy alone for advanced HER2-positive biliary tract cancer) | Very low | **Innovation not assigned** |
| Beremagene geperpavec | Vyjuvek | [Vyjuvek, INN- beremagene geperpavec](https://www.ema.europa.eu/en/documents/assessment-report/vyjuvek-epar-public-assessment-report_en.pdf) | Orphan drug, PRIME - priority medicine, Advanced Therapy | Treatment of patients from birth with dystrophic epidermolysis bullosa (DEB) with mutation(s) in the collagen type VII alpha 1 chain (COL7A1) gene | A | B (The medicine Filsuvez (based on birch bark extract) will be on the market in Europe from 2022) | B (Wound healing significantly superior to placebo (45%, p < 0.001). Small-scale study) | **A -B - B = moderata** | Moderate (There are no approved corrective treatments for DEB patients. For most patients, management is supportive in nature and is mainly limited to palliative care. Current standard of care is focused on reducing trauma and infections while managing the symptoms associated with multiple wounds of varying duration, size, and healing stage, In June 2022, Filsuvez, topical birch bark extract, received marketing authorisation in the EU for the  treatment of dystrophic and junctional EB. ) | Moderate (Study B-VEC-03 (GEM-3) was a multicentre, intrasubject randomised, placebo-controlled, double-blind, phase 3 study of beremagene geperpavec for the topical treatment of DEB wounds. It consisted of a screening phase, followed by a 26-week treatment phase and a 30-day safety follow-up period (SFU). The primary endpoint was complete wound closure at weeks 22 and 24 or weeks 24 and 26; wound closure  between weeks 8 and 12 for the selected wound was considered a key secondary endpoint. Thirty-one subjects were enrolled into the pivotal study, of whom 28 completed it. The efficacy results for the pivotal trial are compelling. In the ITT population, the number of primary wounds  healed at the timepoint of the primary endpoint was 20.9 (67.4%) for the Vyjuvek treated group vs 6.7  (21.6%) for the placebo treated group. There was a significant treatment effect observed between treated  and untreated groups in the ITT population 45.8% p=0.0019.) | Low | **Innovation assigned** |
| Palopegteriparatide | Yorvipath | [Yorvipath, INN-palopegteriparatide](https://www.ema.europa.eu/en/documents/assessment-report/yorvipath-epar-public-assessment-report_en.pdf) | Orphan drug | Parathyroid hormone (PTH) replacement therapy indicated for the treatment of adults with chronic hypoparathyroidism. | A | C3 (Treatment with calcium and vitamin D is already available and, in the most serious cases, recombinant DNA parathyroid hormone) | A (Effective on validated surrogate endpoints) | **A -C3-A = technological, or non-innovation** | Moderate (The goal of current treatment is to relieve symptoms and improve biochemistries (i.e., normalize calcium and phosphate levels) and to prevent neuromuscular sequelae via administration of calcium supplements, active vitamin D, and, in some cases, magnesium supplements if normalization of magnesium levels is indicated. Recombinant human (rh) PTH(1-84) (Natpara/Natpar) was approved as an adjunct to active vitamin D and calcium by the Food and Drug Administration (FDA) in 2015 and by the European Medicines.Approximately 55% of subjects taking Natpar in their pivotal trial were able to reduce conventional therapy by 50% while maintaining serum calcium levels at 7.5 to 10.6 mg/dL (Natpara USPI), thereby partially reducing pill burden; other clinically important changes were not observed. Additionally, Natpar did not reduce the incidence of adverse events (AEs) referable to hypo- or hypercalcemia, and neither normalized nor significantly reduced urinary excretion of calcium) | Minor (The primary efficacy endpoint was to assess the effectiveness of palopegteriparatide on calcium levels, and active vitamin D and calcium levels on week 26. The pivotal TCP-304 Phase III study was a multicentre, randomized, double-blind, placebo-controlled,  parallel group, 26-week trial, with an open-label extension of 3 years of daily palopegteriparatide (TransCon PTH) in male and female adults with chronic hypoparathyroidism of postsurgical,  autoimmune, genetic, or idiopathic etiology for at least 26 weeks, treated with calcitriol (an active vitamin D) ≥0.5 μg/day, or alfacalcidol (an active vitamin D) ≥1.0 μg/day and elemental calcium ≥800  mg/day for at least 12 weeks prior to Screening. Subjects were randomized in a 3:1 ratio into the two treatment group of palopegteriparatide 18 μg/day or placebo for palopegteriparatide (excipient solution), both co-administered with conventional  therapy. A total of 106 subjects were screened and 84 met eligibility criteria. Sixty of 61 treated (63 randomised) subjects in the Verum and 19 of the 21 patients in the Placebo group completed the main study (26 weeks blinded period), indicating a high completion rate.The results of the primary efficacy analysis of the main study showed that the criteria for the composite endpoint were met by significantly more subjects who received palopegteriparatide than subjects who received placebo: 48/61 (78.7% [95% CI: 66.3, 88.1] vs. 1/21 (4.8% [95% CI: 0.1, 23.8]), respectively) | Very low | **Innovation not assigned** |
| Rezafungin | Rezzayo | [Rezzayo Public Assessment Report](https://www.ema.europa.eu/en/documents/assessment-report/rezzayo-epar-public-assessment-report_en.pdf) | Orphan drug [??? Discutibile] | Treatment of invasive candidiasis in adults | A | C1 (other echinocandins are already available i.v., perhaps pharmacokinetic advantage) | B (non-inferiority study with a 20% delta) | **A-C1-B = modest** | Minor (Invasive candidiasis includes both bloodstream and deep-seated invasive infections caused by Candida species. It has already been well established that any delay in initiation of appropriate antifungal therapy results in increased morbidity and mortality. Echinocandins are effective, safe and have very limited drug–drug interactions; however, they require intravenous administration. Also, the current ESCMID guidelines state that oral step-down therapy with fluconazole can be used to simplify treatment if the patient is stable, tolerates the oral route and if the species is susceptible. Other agents used to treat Candida infections include the azoles (fluconazole, itraconazole) and polyenes (amphotericin B products). | Absent (The primary efficacy outcome for the Phase 3 ReSTORE study was global cure (based on clinical cure as assessed by the Investigator, radiological cure [for qualifying IC subjects], and mycological eradication) confirmed by a blinded independent DRC at Day 14 (±1 day). The efficacy of rezafungin in the treatment of candidaemia and invasive candidiasis was considered comparable to caspofungin plus step-down therapy, with regards to global response at Day 14 and ACM at  Day 30. However, the justification for the chosen NIM of -20% is primarily based on ACM as this provides an indication of failure for Global Response, whereas the actual Global Response benefit would be larger than ACM when including the benefit derived regarding non-fatal failures. This margin was discussed with the CHMP /SAWP, it was noted that “meeting a 20% NIM in a single pivotal trial with an observed lower bound of the 95% CI that is >-20% but <-10% could result in an indication that is restricted to patients with limited treatment options”. From the results of the pivotal Phase 3 study, rezafungin was considered non-inferior to caspofungin plus oral step-down therapy in the treatment of patients with candidemia or other forms of IC. However, the chosen non-inferiority margin in this comparative study can be considered as being too wide (20%). Even if the applicant states that although the lower level of the 95% CI resulted in a NI outcome of -14.9%, and that the true benefit of rezafungin to patients is supported by the clear and consistently positive outcomes across a wide range of endpoints, the data is still limited to support that this echinocandin should be considered as  effective as others in the same class. Even if the CI was -14.9, thus very close to -15, the sample size was calculated for a NIM of 20% thus the effect might or might not hold with a different sample size) | Very low | **Innovation not assigned** |
| Trametinib | Spexotras | [Spexotras; INN-trametinib](https://www.ema.europa.eu/en/documents/assessment-report/spexotras-epar-public-assessment-report_en.pdf) | Orphan drug | In combination with dabrafenib is indicated for the treatment of paediatric patients aged 1 year and older with low-grade glioma (LGG) or High-grade glioma (HGG) with a BRAF V600E mutation who require systemic therapy. | A | C1 (vs. conventional treatment with carboplatin + vincristine) | A (more effective than standard treatment) | **A-C1-A = moderate** | Important (Paediatric gliomas constitute approximately 46% of primary brain and other CNS tumours in children and adolescents aged 0-19 years (Ostrom et al 2020). LGGs are rare paediatric tumour types with an incidence of 1.71 cases per 100,000 (Ostrom et al 2020). HGGs are even more rare tumours in the paediatric population, with an incidence of 1.11 cases per 100,000 (Ostrom et al 2020). Approximately 350-400 new cases of paediatric HGG are diagnosed in Europe yearly (EMA 2011). Current therapies for children with HGGs are limited. The present standard of care for newly diagnosed children with HGG is gross total surgical resection, followed by focal irradiation to the tumour bed plus additional chemotherapy (MacDonald 2011). Paediatric patients with LGG harbouring a BRAF V600 mutation seems to have a poorer prognosis than those without this mutation, and require improved treatment options. Currently, temozolomide is the only authorised anticancer substance in EU for paediatric HGG (for use in relapsed or progressive disease), although mostly based on adult efficacy data.) | Moderate (Data to support the sought indications are derived from the multi-centre, open-label, phase II study G2201, conducted in 58 centres across 20 countries. It was a study comprised of two cohorts, to evaluate the effect of dabrafenib in combination with trametinib in paediatric low-grade glioma (LGG cohort), or high-grade glioma (HGG cohort). Overall, the study entry criteria define a population appropriate for the proposed treatment. Moreover, the LGG cohort represents an RCT with a relevant comparator, whereas for the HGG cohort there are no relevant treatment options to which patients could have been randomised. In total, 110 patients entered the LGG cohort and were randomised in a 2:1 ratio to the experimental D+T arm (n=73) or the comparator chemotherapy C+V arm (n=37): A median PFS of 20.1 months (95% CI: 12.8, NE) in the D+T arm versus 7.4 months (95% CI: 3.6, 11.8) in the C+V arm, with HR 0.31 (95% CI: 0.17, 0.55) per Independent review, further supports a clinically relevant efficacy of treatment with D+T in patients with LGG who require first line systemic therapy. The pre-defined success criteria of ORR per Independent review (primary endpoint) in the HGG cohort was met, with a clinically relevant ORR of 56.1%, (95% CI: 39.7, 71.5, 80% CI: 44.9, 66.8). The lower bound of the 95% CI for D+T treatment ORR exceeded the 20% rate prespecified in the study protocol based on the historical rates of ORR for patients with molecularly unselected relapsed refractory HGG (5-12%) treated with standard available therapy.) | Low | **Innovation assigned** |
| Dabrafenib | Finlee | [Finlee; INN-dabrafenib](https://www.ema.europa.eu/en/documents/assessment-report/finlee-epar-public-assessment-report_en.pdf) | Orphan drug | In combination with trametinib is indicated for the treatment of paediatric patients aged 1 year and older with low-grade glioma (LGG) or High-grade glioma (HGG) with a BRAF V600E mutation who require systemic therapy. | A | C1 (vs. conventional treatment with carboplatin + vincristine) | A (more effective than standard treatment) | **A-C1-A = moderate** | Important (Paediatric gliomas constitute approximately 46% of primary brain and other CNS tumours in children and adolescents aged 0-19 years. LGGs are rare paediatric tumour types with an incidence of 1.71 cases per 100,000. HGGs are even more rare tumours in the paediatric population, with an incidence of 1.11 cases per 100,000. Approximately 350-400 new cases of paediatric HGG are diagnosed in Europe yearly (EMA 2011). Current therapies for children with HGGs are limited. The present standard of care for newly diagnosed children with HGG is gross total surgical resection, followed by focal irradiation to the tumour bed plus additional chemotherapy. Paediatric patients with LGG harbouring a BRAF V600 mutation seems to have a poorer prognosis than those without this mutation, and require improved treatment options. Currently, temozolomide is the only authorised anticancer substance in EU for paediatric HGG (for use in relapsed or progressive disease), although mostly based on adult efficacy data.) | Moderate (see above, Study CDRB436G2201: Study of Efficacy and Safety of Dabrafenib in Combination With Trametinib in Paediatric Patients With BRAF V600 Mutation Positive LGG or Relapsed or Refractory HGG Tumors Methods The pivotal study CDRB436G2201 (hereafter referred to as study G2201) combined two paediatric glioma cohorts into a single multi-centre, open-label, phase II study. Patients were enrolled at 58 centers across 20 countries. The two cohorts consisted of paediatric patients with BRAF V600 mutation positive gliomas, with either low-grade glioma (the LGG cohort), or relapsed or refractory high-grade glioma (the HGG cohort). • The LGG cohort was a 2:1 randomized comparison of dabrafenib with trametinib (D+T) versus chemotherapy carboplatin and vincristine (C+V) in chemotherapy naïve LGG patients. • The HGG cohort had a single arm that evaluated the effect of dabrafenib with trametinib (D+T) in relapsed or refractory HGG patients in >2 line.) | Low | **Innovation assigned** |
| Imetelstat | Rytelo | [Rytelo, INN-imetelstat sodium;](https://www.ema.europa.eu/en/documents/assessment-report/rytelo-epar-public-assessment-report_en.pdf) | Orphan drug | Indicated as monotherapy for the treatment of adult patients with transfusion-dependent anaemia due to very low, low or intermediate risk myelodysplastic syndromes (MDS) without an isolated deletion 5q cytogenetic (non-del 5q) abnormality and who had an unsatisfactory response to or are ineligible for erythropoietin-based therapy. | B | B (based on indications, in patients who are unresponsive or unsuitable for erythropoietin) | A (Available clinical data demonstrate clinically relevant efficacy) | **B-B-A = important** | Moderat2 (Myelodysplastic syndromes are among the most common haematologic malignancies in older people, with a median age at diagnosis of over 70 years old and less than 10% of patients being younger than 50 years old. The incidence of MDS increases markedly with age, and the disease is most prevalent in individuals who are white and male. Across identified studies in Europe, incidence of MDS ranged from 0.15 to 0.60 per 10,000 person-years. Currently, allogeneic haematopoietic stem cell transplantation (HSCT) is the only potentially curative therapy for MDS and comes with a high risk of morbidity and mortality, especially in the older MDS patients who often have other complicating comorbidities. In the LR MDS setting, ESA use results in an overall ≥ 8-week TI rate of 20% to 40% and rates tend to be higher in those with low baseline serum erythropoietin (EPO) levels (< 500 U/L, ideally < 100 U/L) and minimal or no RBC transfusion needs. However, responses to ESA therapies tend to be transient (duration of response between 18 and 24 months) and most patients eventually lose response or become refractory.) | Moderate (The multi-centre, placebo-controlled, double-blind, randomised, phase 3 part of study MDS3001 represents the pivotal study for the intended label and the phase 2 part of study MDS3001 provides supportive efficacy and safety results. The study population consisted of patients with IPSS low and intermediate-1 risk MDS. The primary efficacy endpoint was the rate of RBC TI lasting at least 8 weeks. The study was designed to enrol 170 patients. With this sample size, the study was estimated to have 88% power to detect an increase in RBC TI rate from 7.5% with placebo to 30% with imetelstat using a Chi-square test at the 5% level, assuming 10% dropout. Patients were randomly assigned 2:1 to receive either imetelstat or placebo. The primary endpoint, 8-Week Rate of RBC TI, showed statistically significantly higher response rates in the imetelstat treatment group compared with placebo (39.8 and 15.0%; p<0.001). The key secondary endpoint, 24-week rate of RBC TI, showed statistically significantly higher response rates in the imetelstat treatment group compared with placebo (28.0 and 3.3%; p<0.001). | Low | **Innovation assigned** |
| Momelotinib | Omjjara | [Omjjara; INN-momelotinib](https://www.ema.europa.eu/en/documents/assessment-report/omjjara-epar-public-assessment-report_en.pdf) | Orphan drug | Indicated for the treatment of disease-related splenomegaly or symptoms in adult patients with moderate to severe anaemia who have primary myelofibrosis, post polycythaemia vera myelofibrosis or post essential thrombocythaemia myelofibrosis and who are Janus Kinase (JAK) inhibitor naïve or have been treated with ruxolitinib. | B [riferita alla splenomegalia associata alla mielofibrosi, non alla malattia di base] | B [è una alternativa al ruxolitinib] | B [efficacia meaningful sulla splenomegalia, ma non sulla mielofibrosi in quanto tale] | **B-B-B = moderate** | Moderato (Myelofibrosis (MF) is a rare Philadelphia chromosome (Ph1)-negative myeloproliferative neoplasm (MPN) that may occur de novo as primary MF (PMF) or as secondary MF (SMF) when it evolves from other pre existing MPNs polycythaemia vera (PV) or essential thrombocythemia. MF has an incidence of about 0.58 new cases per 100,000 person-years, but a higher prevalence of 6 per 100,000 person-years because of its chronic and disabling course. Allogeneic HSCT is the only curative therapy for MF (Robin, 2019). However, the procedure is associated with high morbidity and mortality, particularly in older adults, and is thus generally considered for only a limited subset of patients aged < 70 years with suitable donors, lack of significant comorbidities, and good performance status (Tiribelli, 2020). For many patients who are ineligible for allogeneic HSCT, the current standard of care includes treatment with an approved JAK inhibitor: Ruxolitinib, Fedratinib. Other treatments. Before JAK inhibitors were available for MF, treatment options were limited. Hydroxycarbamide (hydroxyurea). | Moderato (The efficacy assessment of momelotinib in patients with moderate to severe anaemia (Hgb < 10 g/dL, as per eligibility criteria) in patients previously treated with ruxolitinib is mainly based on the results of the MOMENTUM trial. In the JAK inhibitor naïve setting, the efficacy of momelotinib in patients with moderate to severe anaemia is mainly based on post-hoc analysis in the subgroup of patients with Hgb. In patients that have been treated with ruxolitinib: - MOMENTUM with 24 weeks randomized, double-blind treatment period and open-label extended treatment phase (up to 180 weeks) with momelotinib treatment in both arms. Danazol-treated subjects who did not cross-over to momelotinib at Week 24 had the option to continue danazol as open-label treatment through week 48. SIMPLIFY-1 with 24 weeks randomized, double-blind period and up to 5 years open-label, with momelotinib monotherapy after 24 weeks in both arms. Reduction of spleen volume (assessed by splenic response rate) and improvement of disease-related symptoms (assessed by total symptom score response rate) has been assessed as primary or key secondary endpoints. Both objectives are related to each other (i.e. reduction of spleen volume is associated with improvement of symptoms). The MOMENTUM trial met its first primary efficacy endpoint, demonstrating statistically significant superior MFSAF TSS response rate at Week 24 for MMB over DAN [24.62% (95% CI: 17.49, 32.94) versus 9.23% (95% CI: 3.46, 19.02); p = 0.0095]. In the supportive SIMPLIFY-2 study, despite lack of a washout period, despite the differences in the used PRO instruments and despite the uncertainty in this endpoint due to the open-label design of this study, the Week 24 modified MPN-SAF v2.0 TSS response rate observed in the SIMPLIFY-2 study supports the effect of MMB on symptom response observed in the MOMENTUM study, with a similar TSS response rate at Week 24 of 26.2% (95% CI: 18.04, 35.80), which was numerically higher compared to the BAT control arm [5.9% (95% CI: 1.23, 16.24); p < 0.001]. | Low | **Innovation assigned** |
| Rozanolixizumab | Rystiggo | [Rystiggo- INN-rozanolixizumab](https://www.ema.europa.eu/en/documents/assessment-report/rystiggo-epar-public-assessment-report_en.pdf) | Orphan drug | Indicated as an add-on to standard therapy for the treatment of generalised myasthenia gravis (gMG) in adult patients who are anti-acetylcholine receptor (AChR) or anti-muscle-specific tyrosine kinase (MuSK) antibody positive. | A | B (as an add-on therapy) | C (the therapeutic effect lies in the reduction of symptoms) | **A-B-C = modest** | Moderate (Myasthenia gravis most commonly affects young adult women (aged under 40 years) and older men (aged over 60 years), but it can occur at any age. Information from Orphanet suggests that the prevalence of MG in the EU is estimated to be 1 per 5,000 population. First-line treatment for MG is symptomatic with acetylcholinesterase (AChE) inhibitors such as pyridostigmine bromide. This and other AChE inhibitors directly increase the concentration of acetylcholine at the NMJ and thereby improve signal transmission through increased engagement of postsynaptic acetylcholine receptors. Immunosuppressive treatment of gMG is dominated by untargeted treatments such as steroids and nonsteroidal immunosuppressants (e.g, azathioprine). Both steroids and nonsteroidal immunosuppressants target the immune system non-specifically with the goal of reducing autoimmune reactivity in MG. Rituximab, an anti-CD20 monoclonal antibody (mAb) is reserved for patients that are refractory to conventional oral immunosuppressants and used as part of an escalation therapy). | Minor (The evaluation of efficacy is primarily based on the single pivotal, placebo-controlled Phase 3 study (MG0003) in moderate to severe gMG with four flat SC dosing regimens in ≈7mg/kg (and ≈10mg/kg treatment arms, based on four weight tiers. Study MG0003 does not address sustained efficacy beyond the first cycle or after frequent re administration. Additional data from the interim cutoff of the ongoing Phase 3 OLE study (MG0007, repeated cyclic treatment as clinically needed) as well as the completed Phase 3 OLE study (MG0004, chronic treatment) provided evidence to support the safety and efficacy evaluation for rozanolixizumab beyond first treatment cycle. Overall, 128 (64%) patients completed the study and 52 (81.3%) patients in the rozanolixizumab ≈7mg/kg group, 48 (69.6%) in the ≈10mg/kg group, and 56 (83.6%) in the placebo group received all 6 infusions (excluding mock infusions). The evaluation of efficacy was based mainly on MG-ADL scale (subjective assessment of MG symptoms by the patient) and supported by QMG scale (quantitative evaluation of relevant muscle groups by the physician), MGC scale (combining physician examination and patient reported outcomes) and MG symptoms PRO score (which was developed by the applicant). MG0003 study met its primary endpoint, meaning symptomatic relief on MG-ADL as reported by patients (less functional disability of MG patients), during first treatment cycle (RS). At Day 43 (Visit 10), the Assessment report EMA/CHMP/535512/2023 Page 149/217 primary endpoint was statistically significant for both rozanolixizumab treatment groups. The differences in least square (LS) mean change from Baseline in MG-ADL score between groups (rozanolixizumab minus placebo) were -2.586 (95% CI: -4.091, -1.249). | Very low | **Innovation not assigned** |
| Elafibranor | Iqirvo | [Iqirvo, INN-elafibranor](https://www.ema.europa.eu/en/documents/assessment-report/iqirvo-epar-public-assessment-report_en.pdf) | Conditional approval, Orphan drug | Indicated for the treatment of primary biliary cholangitis (PBC) in combination with ursodeoxycholic acid (UDCA) in adults with an inadequate response to UDCA, or as monotherapy in patients unable to tolerate UDCA. | A | B (as an add-on to ursodeoxycholic acid or as an alternative to the latter if not tolerated) | C (favorable results on biochemical endpoints, but the normalization of ALP, which would be the optimal goal to achieve, although a clear predictive value on long term outcome is not established, it is seen only in a limited subset of patients (roughly 15%).) | **A-B-C = modest** | Minor (The estimated prevalence of primary biliary cholangitis (PBC) in North America, Europe, and the Asia-Pacific region varies from 1.91 to 40.2 per 100,000 persons. The only approved medical therapies in the United States (US) and Europe for the treatment of PBC are UDCA. UDCA at a daily dose of 13 to 15 mg/kg/day is the only approved first-line therapy for the treatment of PBC [EASL 2017; Lindor 2019]. Treatment with UDCA has been shown to improve liver biomarkers of response, slow disease progression, and improve transplant-free survival [Corpechot 2011]. However, up to 40% of patients treated with UDCA have an inadequate response, and such patients remain at high risk of disease progression and have reduced transplant-free survival rates compared to those classified as UDCA responders. | Moderate (The pivotal Study 319-1 was a multicentre, multinational, randomised (2:1), double-blind (DB), placebo-controlled, parallel-group phase III study followed by an open-label LTE evaluating the efficacy and safety of elafibranor 80 mg die in participants with PBC with inadequate response or intolerance to UDCA. Overall, 108 participants were included in the elafibranor ITT Analysis Set, and 53 participants were included in the placebo ITT Analysis Set. The primary endpoint was the response to treatment at week 52, defined as a composite biochemical endpoint i.e. ALP < 1.67 that, although not validated, is an intermediate measure of effect used in PBC setting and reported in current EMA GL. The actual clinical relevance of such endpoint and the predictive value on clinical benefit remains to be demonstrated for elafibranor. Regarding the primary endpoint (composite biochemical endpoint i.e. ALP, at Week 52, the proportion of responders was 55/108 (50.9%) participants in the elafibranor group compared with 2/53 (3.8%) participants in the placebo group, resulting in a statistically significant estimated difference of 47.2% (95% CI: 32.0; 56.9) favouring the elafibranor group. The estimated OR (elafibranor versus placebo) was statistically significant in favour of elafibranor (OR=37.6 [95% CI: 7.6; 302.2]; p<0.0001). | Very low | **Innovation not assigned** |
| Serplulimab | Hetronifly | [Hetronifly, INN-serplulimab](https://www.ema.europa.eu/en/documents/assessment-report/hetronifly-epar-public-assessment-report_en.pdf) | Orphan drug | In combination with carboplatin and etoposide is indicated for the first-line treatment of adult patients with extensive-stage small cell lung cancer (ES-SCLC). | A | B (There are already other medicines available with a similar indication but this is in addition to the first line) | B (OS increase of just over 4 months compared to the comparison group) | **A-C-B = moderate** | Moderate (Extensive stage-small cell lung cancer represents about 15% of all lung cancers (Wéber et al 2023). Most cases of SCLC present as extensive-stage (~70%), while the remaining (~30%) present with limited disease. In general, surgery is the only therapeutic treatment in SCLC, but it is no option in ES-SCLC. Standard of care since the 1990s has been platinum-based chemotherapy regimen including two agents, either cisplatin or carboplatin in combination with etoposide. The 2021 ESMO guideline recommendations for SCLC include cisplatin or carboplatin in combination with etoposide in ES-SCLC, in addition to atezolizumab and durvalumab as induction and maintenance.) | Important (The pivotal study (ASTRUM-005) was a phase III, randomised, double-blind and placebo-controlled trial. The objective of the study was to compare the efficacy and safety of serplulimab in combination with platinum-based chemotherapy versus placebo in combination with platinum-based chemotherapy in treatment-naïve patients with ES-SCLC. The ASTRUM-005 trial was designed to show superiority using 2:1 allocation of patients to the serplulimab and placebo groups. A total of 585 subjects were included. The primary endpoint OS is a valid and reliable measure of clinical benefit in the intended patient population. The median OS for the overall population was 15.4 months in the serplulimab group and 10.9 months in the placebo group, with HR (95% CI) of 0.63 (0.49, 0.82). The increase in median OS of 4.5 months in the serplulimab group is considered clinically meaningful for the intended population.) | Moderate | **Innovation assigned** |
| linvoseltamab | Lynozyfic | [Lynozyfic- INN, linvoseltamab](https://www.ema.europa.eu/en/documents/assessment-report/lynozyfic-epar-public-assessment-report_en.pdf) | Conditional approval | Indicated as monotherapy for the treatment of adult patients with relapsed and refractory multiple myeloma who have received at least 3 prior therapies, including a proteasome inhibitor, an immunomodulatory agent, and an anti-CD38 monoclonal antibody, and have demonstrated disease progression on the last therapy. | A | B (drug for second-third line treatment) | C (The CHMP deemed the PFS data insufficient for full approval and gave conditional approval pending further evidence by 2027) | **A-B-C = modest** | Important (Novel treatments introduced in the last 20 years have improved life expectancy for patients with MM, and the estimated 5-year survival rate now exceeds 50% . However, despite significant therapeutic advances, approximately 10% of patients die within 1 year of diagnosis, and early deaths due to disease progression or treatment toxicity are still a clinically relevant issue, with 6% and 24% of patients experiencing fatal events before or during their first-line treatment, respectively . Real-world data also suggest that approximately 40% and 62% of patients are not able to proceed to 2nd and 3rd line treatment, respectively ). Several compounds are approved in the EU for the treatment of MM, yet immunomodulators (IMiDs, e.g. lenalidomide, thalidomide and pomalidomide), proteasome inhibitors (PIs, e.g. bortezomib, carfilzomib and ixazomib) and anti-CD38 monoclonal antibodies (anti-CD38 mAbs, e.g. daratumumab, isatuximab) form the backbone of most regimens indicated for the treatment of NDMM and R/R MM.) | Minor (Phase 2 was designed to investigate the safety and activity of linvoseltamab in advanced settings of RR MM, and two different recommended Phase 2 doses (RP2Ds 50 mg and 200 mg) were selected for further testing in dedicated cohorts. The primary efficacy objective in the Phase II part of study 1826 was to evaluate the anti-MM activity of linvoseltamab in terms of objective response rate (ORR, i.e. subjects with a best overall response of PR, VGPR, CR or sCR), as assessed by an independent review committee (IRC). At the time of the data cutoff date of the primary analysis (DCO 08 Sept 2023), a total of 349 patients were screened for study participation and 282/349 (~80%) were enrolled and treated in pivotal study 1826. Study 1826 met its primary objective: the Objective response rate (ORR) by IRC in the Phase 2 200 mg cohort in the most updated analysis was 69.5% (95%CI 59.8, 78.1), which significantly exceeded the pre-specified threshold for efficacy (31%). | Very low | **Innovation not assigned** |
| Sparsentan | Filspari | [Filspari, INN-sparsentan](https://www.ema.europa.eu/en/documents/assessment-report/filspari-epar-public-assessment-report_en.pdf) | Conditional approval, Orphan drug | Indicated for the treatment of adults with primary immunoglobulin A nephropathy (IgAN) with a urine protein excretion ≥1.0 g/day (or urine protein-to-creatinine ratio ≥ 0.75 g/g. | A | B (other drugs of the renin-angiotensin system are already available, and this one claims an additional mechanism of action, endothelin type A receptor antagonist). | B (Due to insufficient evidence of long-term therapeutic effect, the CHMP gave conditional approval) | **A-B-B = moderate** | Moderate (Immunoglobulin A Nephropathy (IgAN), categorised as a rare disease affecting the kidneys, is a serious, progressive, and life-limiting disease with a poor prognosis and high unmet medical need in Europe and worldwide. There are limited approved treatments for IgAN in the EU. The current treatment strategy is aimed at preventing or delaying ESKD. The main clinical predictor of disease progression in patients with IgAN is proteinuria. Standard-of-care treatment for IgAN patients consists of renin-angiotensin-aldosterone system (RAAS) inhibitors (angiotensin-converting enzyme inhibitors (ACEIs) or angiotensin II receptor blockers [ARBs]) to reduce proteinuria and manage blood pressure, along with supportive interventions such as dietary and lifestyle modifications (KDIGO 2021). | Moderate (Clinical efficacy data was collected mainly from the phase 3 trial (PROTECT). It is a randomised, multicenter, double-blind, parallel-group, active-control study in subjects with IgAN, followed by an OLE phase trial. Irbesartan was chosen as an active comparator. The primary endpoint of change in baseline urinary protein/creatinine ratio in 24 hours per week is an acceptable biomarker to evaluate the early onset of effect within the context of IgAN. In IgA nephropathy, proteinuria is believed to be in the disease pathway toward kidney damage, and the marker is used in clinical practice to monitor disease severity (episodes) and treatment success. A total of 406 patients enrolled in the PROTECT study, of which 202 were randomised to sparsentan and 202 to irbesartan. The primary efficacy endpoint in the interim analysis shows superiority over irbesartan in reducing proteinuria at week 36 of treatment. The results of the primary efficacy endpoint are clinically and statistically significant. Proteinuria change may be acute and reversible vs chronic and persistent. However, this needs to be confirmed in the final CSR (Specific obligation, Annex II.E). Although only limited number of patients > 65 years were included, results were consistent with overall data. Sensitivity analyses showed consistency with the primary effect. No dose adjustment is necessary in the elderly, as reflected in the SmPC | Low | **Innovation assigned** |
| Iptacopan | Fabhalta | [Fabhalta, INN - iptacopan](https://www.ema.europa.eu/en/documents/assessment-report/fabhalta-public-assessment-report_en.pdf) | Orphan drug, PRIME - priority medicine | Indicated as monotherapy in the treatment of adult patients with paroxysmal nocturnal haemoglobinuria (PNH) who have haemolytic anaemia. | A | C1 (There are other drugs available but each of these leaves some patients not fully responsive) | B (effective on validated surrogate endpoints, haemoglobin and fatigue, however it does not alter the natural history of the disease) | **A-C1-B = modest** | Minor (Paroxysmal nocturnal haemoglobinuria (PNH) has an annual incidence of 1-10 new cases per 1 million individuals. The C5 inhibitors eculizumab (Soliris) and ravulizumab (Ultomiris) have shown increased survival and improved outcomes in PNH by controlling IVH, reflected in LDH improvements; however, C5 inhibitors do not control EVH. In many patients treated with C5 inhibitors, although LDH is largely controlled, ARC and bilirubin levels remain elevated, indicative of ongoing haemolysis. Despite the availability of eculizumab over the past 13 years, some patients remain symptomatically limited by their disease and still require PRBC transfusion because not all key markers of disease activity are meaningfully improved. Danicopan (Voydeya), a reversible inhibitor of complement factor D, was recently approved as an add-on to ravulizumab or eculizumab for the treatment of adult patients with PNH who have residual haemolytic anaemia. Pegcetacoplan (Aspaveli), targeting C3 in the proximal complement system, was approved in 2021 for patients who are anaemic after treatment with a C5 inhibitor for at least 3 months. Pegcetacoplan (Aspaveli), targeting C3 in the proximal complement system, was approved in 2021 for patients who are anaemic after treatment with a C5 inhibitor for at least 3 months. Pegcetacoplan was recently approved in the EU as monotherapy for the treatment of adult patients with PNH who have haemolytic anaemia) | Moderate (APPLY-PNH is a randomised, open-label, active-comparator trial which enrolled 97 adult PNH patients with residual anaemia (Hb < 10 g/dl) despite receiving a stable regimen of anti-C5 antibody therapy (eculizumab or ravulizumab) for at least 6 months prior to randomisation. Patients received iptacopan monotherapy 200 mg b.i.d. (n=62) or continued their anti-C5 regimen (n=35) for 24 weeks (randomised treatment period, RTP). Anti-C5 therapy with the monoclonal antibodies eculizumab or ravulizumab is considered standard of care for haemolytic PNH and were the only approved PNH therapy at time of study initiation. Albeit it was recommended against open-label conduction in a SA (EMEA/H/SA/3968/3/2019/III), the open-label design seems acceptable, as Hb as an important component of the primary endpoint was objectively measured. The primary efficacy analysis was based on two haematological responder endpoints: - Increase from baseline Hb levels > 2 g/dl. Iptacopan monotherapy 200 mg bid was statistically significantly superior to anti-C5, with an estimated treatment difference (95% CI) in the primary endpoint of: 80.3% (71.3 (71.2)1, 87.6) for Hb increase > 2 g/dl and 67.0% (56.3 (56.4)1, 76.9) for Hb > 12 g/dl from baseline in the absence of transfusion. The APPLY results demonstrated superiority of iptacopan monotherapy over anti-C5 therapy across a broad spectrum of clinically relevant endpoints in haemolytic PNH over the 24-week RTP in terms of clinically meaningful increases in haemoglobin levels, significant reduction of the need for RBC transfusions and meaningful improvements of patient-reported fatigue. | Moderate | **Innovation not assigned** |
| Zolbetuximab | Vyloy | [Vyloy, INN-zolbetuximab](https://www.ema.europa.eu/en/documents/assessment-report/vyloy-epar-public-assessment-report_en.pdf) | Orphan drug | In combination with fluoropyrimidine- and platinum-containing chemotherapy, is indicated for the first line treatment of adult patients with locally advanced unresectable or metastatic HER2 negative gastric or gastro-oesophageal junction (GEJ) adenocarcinoma whose tumours are Claudin (CLDN) 18.2 positive. | A | B (first-line treatment in combination with other chemotherapy drugs) | B (effective on PFS and OS, but with doubts about its efficacy in subgroups of patients) | **A-B-B = moderate** | Moderate (CLDN18.2 is expressed in a number of human cancers including gastric and pancreatic adenocarcinomas [Lee et al, 2011]. The expression of CLDN18.2 is retained upon malignant transformation of gastric epithelia and is present in approximately 80% of primary gastric adenocarcinomas. CLDN18.2 expression was detected in diffuse and intestinal gastric adenocarcinomas. Currently recommended first-line therapies for locally advanced unresectable or metastatic disease include chemotherapy backbone (fluoropyrimidine- and platinum-containing cytotoxic drugs) in combination with therapy depending on HER2 and PD-L1 CPS status. For the 80% of patients with HER2-negative locally advanced or metastatic gastric/GEJ adenocarcinoma, the treatment is chemotherapy alone or, based on PD-L1 CPS status, in combination with nivolumab or pembrolizumab. OPDIVO was approved in 2021 (Opdivo II/96) in combination with fluoropyrimidine- and platinum-based combination chemotherapy for the first-line treatment of adult patients with HER2-negative advanced or metastatic gastric, gastrooesophageal junction or oesophageal adenocarcinoma whose tumours express PD-L1 with a combined positive score (CPS) ≥ 5. Keytruda received an approval in November 2023 (Keytruda II/135) for the first-line treatment of HER2-negative gastric or gastro-oesophageal junction adenocarcinoma in adults whose tumours express PD L1 with a CPS ≥ 1.) | Moderate (Two global, randomized, double-blind and placebo-controlled phase 3 studies evaluated zolbetuximab in combination with fluoropyrimidine- and platinum containing chemotherapy as first line treatment in participants with locally advanced unresectable or metastatic HER2 negative gastric (GC) or gastroesophageal junction (GEJ) adenocarcinoma whose tumors were CLDN18.2-positive. Participants in the SPOTLIGHT study received zolbetuximab or placebo in combination with mFOLFOX6 (n=565); the GLOW study evaluated zolbetuximab plus CAPOX compared with placebo plus CAPOX (n=507). Duration of therapy was “until disease progression or unacceptable toxicity”. The primary endpoint was PFS assessed by independent review (IRC), with OS as key secondary endpoint. Study SPOTLIGHT reported a PFS HR of 0.75 (95% CI 0.598, 0.942), a difference in median PFS of 1.9 months, and a 10% PFS rate difference at 18 months. In study GLOW, the PFS HR was 0.687 (95% CI 0.544, 0.866) and the difference in median PFS was 1.4 months with a 19% PFS rate difference at 12 months (difference 13% at 18 months with low numbers at risk). The survival benefit in study SPOTLIGHT was based on a HR of 0.75 (95% CI 0.6, 0.94), a 2.7 months improvement of median OS and a 10% OS rate difference at 24 months. Study GLOW reported an OS HR of 0.771 (95% CI: 0.615, 0.965), a 2.2 months improvement of median OS and a 10% OS rate difference at 18 months. The information fraction was 82% for both SPOTLIGHT and GLOW. Of note, OS results did not seem to have been impacted by differences in post-progression therapies. Most frequent post-progression therapies were paclitaxel (17% vs 19.5% and 18.1% vs 20.2% for zolbetuximab vs placebo in SPOTLIGHT and GLOW) and ramucirumab (12.4% vs 12.1% and 8.3% vs 11.1% for zolbetuximab vs placebo in SPOTLIGHT and GLOW). | Very low | **Innovation assigned** |
| Elranatamab | Elrexfio | [ELREXFIO, INN-elranatamab](https://www.ema.europa.eu/en/documents/assessment-report/elrexfio-epar-public-assessment-report_en.pdf) | Conditional approval, PRIME - priority medicine | Indicated as monotherapy for the treatment of adult patients with relapsed and refractory multiple myeloma, who have received at least three prior therapies, including an immunomodulatory agent, a proteasome inhibitor, and an anti-CD38 antibody and have demonstrated disease progression on the last therapy. | A | B (indicated after three lines of treatment) | C (partial results, to be confirmed) | **A-B-C = modest** | Important (The treatment landscape for relapsed or refractory multiple myeloma (RRMM) has changed in recent years. Current treatment of MM includes glucocorticoids, chemotherapy, primarily alkylating agents, high dose chemotherapy followed by ASCT, proteasome inhibitors (PIs, such as bortezomib, carfilzomib and ixazomib), immunomodulatory agents (such as thalidomide, lenalidomide and pomalidomide), monoclonal antibodies ((mAbs), such as daratumumab, isatuximab and elotuzumab) and the histone deacetylase inhibitor panobinostat. Common standard regimens include either a PI or an IMiD in combination with dexamethasone with or without a monoclonal antibody such as daratumumab. The triplet combination of bortezomib, lenalidomide, and dexamethasone (VRd) is a standard. Newer classes of medications including XPO1 inhibitors (selinexor) and antibody drug conjugates targeting BCMA (belantamab mafodotin-blmf) and also CAR T cell products (Idecaptagene vicleucel, Cilacaptagene autoleucel) have been approved by EMA. The latest was talquetamab, which is a bsAb that targets GPRC5D and CD3.) | Minor (In support of this application efficacy results from the single arm trial C10071003, which is being conducted in 10 countries in Europe, North America, and Japan have been submitted. The target population of this phase 2 study represents a MM patient population with multi-refractory disease where most patients were previously treated with several (median 5) previous lines of therapies. The pivotal Cohort A in this study included 123 participants who had received at least one PI, one IMiD, and one anti-CD38 mAb and who were relapsed or refractory to the last anti-MM regimen and naïve to prior BCMA-directed therapy. Enrolled participants received SC elranatamab with a 2 step-up priming regimen of 12 mg on C1D1 and 32 mg on C1D4 followed by the first full dose (76 mg) of elranatamab on C1D8 and QW thereafter, except for the first 4 participants that received 1 step-up priming dose of 44 mg on C1D1 followed by the first full dose (76 mg) on C1D8. If a participant received QW dosing for at least 6 cycles and achieved an IMWG response category of PR or better persisting for at least 2 months, the dose interval was to be changed from QW to Q2W (e.g., beginning C7D1). The primary objective of the study was to determine the efficacy of elranatamab. The corresponding endpoint was confirmed ORR by BICR per IMWG. The primary endpoint was ORR by BICR which was 61.0% (95% CI: 51.8, 69.6; null rate of 30%). 35.8% (95&CI: 27.3, 44.9) achieved CR or better. That means that the treatment is efficacious but as this study is a SAT this is a limitation for granting full approval. | Low/very low | **Innovation not assigned** |
| Sotatercept | Winrevair | [WINREVAIR, INN-sotatercept](https://www.ema.europa.eu/en/documents/assessment-report/winrevair-epar-public-assessment-report_en.pdf) | Orphan drug, PRIME - priority medicine | In combination with other pulmonary arterial hypertension (PAH) therapies, is indicated for the treatment of PAH in adult patients with WHO Functional Class (FC) II to III, to improve exercise capacity | A | C2 (The pivotal study STELLAR demonstrated that treatment with sotatercept resulted in a significant improvement in 6MWD from baseline to week 24 of 40.8 m (p< 0.001), which is considered clinically relevant since it is in the range observed with other approved PAH therapies) | C | **A-C2-C = pharmacological, or non-innovation** | Moderate (Pulmonary arterial hypertension (PAH) is a rare (15 to ~60 people per million), chronic, progressive, and fatal disease that can present at any age, causing limitations in physical activity and quality of life despite treatment with approved therapies. Current PAH therapies improve exercise capacity and prolong time to clinical worsening but have not shown an effect on overall mortality. Approved therapies act via the prostacyclin, endothelin, or nitric oxide pathways, believed to primarily mediate their effects through pulmonary vasodilation. Current PAH therapies include agents such as ERAs, PDE5 inhibitors, soluble guanylate cyclase stimulators, and/or prostacyclin analogues or receptor agonists, in addition to general supportive care agents (e.g. anticoagulants, diuretics, digoxin). Sotatercept is an activin signaling inhibitor with high selectivity for activin-A, a dimeric glycoprotein which belongs to the TGF-β superfamily of ligands. Activin-A binds to ActRII Assessment report Page 13/217 (both ActRIIA and ActRIIB) regulating key signaling for inflammation, cell proliferation, apoptosis, and tissue homeostasis.) | Minor (The design of STELLAR generally appeared appropriate. The study consisted of four periods, including a screening period, double-blind placebo-controlled (DBPC) treatment period, a long-term double-blind treatment period and a follow-up period. In the double-blind placebo-controlled (DBPC) period of 24 weeks, 323 eligible subjects were randomized (1:1) to receive either sotatercept or placebo subcutaneous Q3W in addition to background PAH therapy. The starting dose of sotatercept was 0.3 mg/kg at Visit 1, followed by 0.7 mg/kg Q3W for the remainder of the study. The primary endpoint of change from baseline in 6MWT after 24 weeks is considered appropriate for the indication ‘to improve exercise capacity’, since this is in line with CHMP guideline on the clinical investigations of medicinal products for the treatment of pulmonary arterial hypertension. In the primary efficacy analysis, treatment with sotatercept resulted in a significant improvement in 6MWD from baseline to week 24 as compared to placebo in patients with PAH (WHO Group 1) on background PAH therapy (median: 34.4 m vs. 1.0 m, respectively; median treatment difference (Hodges-Lehmann location shift) of 40.8 m (95% CI : 27.5, 54.1); p< 0.001) from baseline levels of 397.6 m and 404.7 m for the sotatercept and placebo group, respectively. This increase is considered clinically relevant as it compares to the results of the pivotal study of already authorised products for PAH.) | Moderate | **Innovation not assigned** |
| Vamorolone | Agamree | [Agamree, INN-vamorolone](https://www.ema.europa.eu/en/documents/assessment-report/agamree-epar-public-assessment-report_en.pdf) | Orphan drug | Indicated for the treatment of Duchenne muscular dystrophy (DMD) in patients aged 4 years and older. | A | C3 (It has essentially a glucocorticoid effect, similar to drugs in this class already used in DMD. Therefore, it is a new molecule with a therapeutic role similar to that of other corticosteroids) | C (It produced symptomatic effects similar to those of prednisone. The safety profile was also similar to that of prednisone) | **A-C3-C = technological, or non-innovation** | Important (Duchenne muscular dystrophy (DMD) in patients aged 4 years and older is a severe, progressive paediatric neuromuscular disorder that is ultimately lethal. It occurs almost exclusively in males (X-linked recessive disorder) with an estimated male birth prevalence of 1/3,500- 1/9,300. The estimated prevalence in the EU is approximately 15,000 cases. No medical cure exists for DMD, and the disease has a poor prognosis. The main aim of DMD treatment is the modification of the natural course of the disease or prolonging survival. Treatment is centred on glucocorticoid therapy, prevention of contractures, and medical care of cardiomyopathy and respiratory compromise (Shimizu-Motohashi 2019; McMillan 2019). Glucocorticoids should be continued after loss of ambulation according to current guideline recommendations (Birkrant et al. 2018) and have been shown to improve strength and motor function, delay loss of ambulation by 2 to 3 years, preserve upper limb and respiratory function, avoid scoliosis surgery, and delay the onset of cardiomyopathy. Recent studies confirm the benefits of starting glucocorticoids in younger children, before significant physical decline. For a subpopulation of DMD patients, in which mutation created a nonsense stop codon in the dystrophin mRNA resulting in premature termination of translation and, hence, a truncated, non-functional protein, ataluren (Translarna) was granted a conditional marketing authorisation in the EU on 31 July 2014 (EMEA/H/C/2720) for the treatment of patients aged 5 years and above, which has been extended to ambulatory patients aged 2 years and older. Thus, there is an unmet medical need for mutation-independent treatments similar to glucocorticoids with an acceptable benefit-risk profile. Vamorolone is a synthetic corticosteroid analogue that structurally diverges from other members of the class of glucocorticoid agents.) | Minor (Pivotal Study VBP15-004: A total of 121 subjects were randomised with 30 subjects in each of the vamorolone treatment arms and the combined placebo groups and 31 in the combined prednisone groups. According to the study protocol patients had to have a confirmed diagnosis of DMD, either by genetic testing or based on muscle biopsy. While almost all patients were diagnosed based on genetic confirmation by DNA and had no additional muscle biopsy, there were three subjects solely diagnosed based on muscle biopsy. Genetic mutations were similar across the study arms. In the primary analysis of change from baseline in TTSTAND velocity (Time to stand (velocity)) at week 24, a statistically significant difference was demonstrated when vamorolone 6 mg/kg/day was compared against placebo (p = 0.0018). TTSTAND velocity increased in the vamorolone 6 mg/kg group from 0.186 to 0.242 rises/sec and slightly decreased in the placebo group from 0.200 to 0.193 rises/sec at Week 24. The LSM difference for vamorolone 6 mg/kg from placebo was 0.0586 rises/sec in the EMA analysis and 0.0603 rises/sec in the primary FDA analysis. The improvement in TTSTAND velocity under vamorolone 6 mg/kg/day is considered clinically relevant as it is greater than the MCID for TTSTAND of 0.023 rises/sec. TTCLIMB Velocity was increased in each of the vamorolone groups and slightly decreased in the placebo group from baseline at Week 24. The LSM difference was 0.07 tasks/sec for vamorolone 6 mg/kg from placebo (p= 0.0005 and p = 0.0008, respectively) and 0.06 tasks/sec for vamorolone 2 mg/kg from placebo in the EMA and the FDA analyses (p= 0.005 and p = 0.0056, respectively). The improvements in TTCLIMB velocity with vamorolone 2 and 6 mg/kg were both greater than the MCID. Effects of the primary analyses were corroborated by a number of sensitivity and supportive analyses of the primary endpoint as well as secondary endpoints evaluating motor function in Duchenne. No loss of efficacy was seen when subjects were switched from prednisone to vamorolone 6 mg/kg at Week 24, whereas small but also inconsistent declines were seen after the switch from prednisone to vamorolone 2 mg/kg.) | Moderate | **Innovation not assigned** |
| Omaveloxolone | Skyclarys | [Skyclarys, INN-omaveloxolone](https://www.ema.europa.eu/en/documents/assessment-report/skyclarys-epar-public-assessment-report_en.pdf) | Orphan drug | The treatment of Friedreich’s ataxia in adults and adolescents aged 16 years and older. | A | A | C (a limited study, on a case study excluding the most serious, compared with a placebo, appears to reduce the progression of the disease) | **A-A-C = moderate** | Important (Friedreich’s ataxia (FA) is a rare, genetic, life-shortening, debilitating, and degenerative neuromuscular disorder. In a 2013 study, Vankan synthesised available epidemiology studies that focused on different regions in Europe and concluded that approximately 2 in 100,000 people are affected by FA in Europe. Currently, there are no approved therapies for FA. The most recent consensus clinical management guidelines for FA recommend symptomatic treatment only. The standard of care for FA is similar globally and includes palliative and symptomatic treatments. Medications such as iron chelators, vitamin supplements, and frataxin level modifiers) | Minor (Omaveloxolone is an orally bioavailable triterpenoid analogue and a potent activator of the transcription factor, Nrf2. Omaveloxolone selectively and reversibly binds to Keap1 (Kelch-like ECH-associated protein 1), allowing for nuclear translocation of Nrf2 and transcription of its target genes. Nrf2 controls the expression of genes involved in mitochondrial function, redox balance, and inflammation. Omaveloxolone has been developed for the treatment of FA in adults and adolescents aged 16 years and older. Phase II Study 408-C-1402 enrolled a total of 172 patients overall (33 adolescent patients) and provides all efficacy and safety data for omaveloxolone with the longest treatment duration of 3.7 years. This study was conducted in 3 parts: Study 1402 Part 1 (dose finding, n=69), Study 1402 Part 2 (pivotal, n=103) and Study 1402 Extension (open-label extension, n=149, interim analysis data cutoff date 24 March 2022). Study 1402 Part 2 was an international, multicentre, double-blind, randomised, placebo-controlled, registrational, parallel-group phase 2 trial to evaluate the safety and efficacy of omaveloxolone 150mg per day in patients with FA. The key efficacy analyses were based on change from baseline to week 48 in mFARS in patients without pes cavus. FARS is a well validated and known scale used by specialists as a series of physical examination assessments to measure impairment level, functional status and disease progression in patients with FA. 156 patients were screened and a total of 103 patients were randomised into Study 1402 Part 2; 51 patients to omaveloxolone and 52 patients to placebo. A total of 44 (86.3%) omaveloxolone-treated patients and 50 (96.2%) placebo-treated patients completed treatment through Week 48; of these, respectively, 41 and 34 patients had mFARS assessed at Week 48. The study met its primary efficacy endpoint: omaveloxolone significantly improved mFARS relative to placebo (mean difference of -2.40, p = 0.014) after 48 weeks. The key secondary endpoints did not formally contribute to evidence for efficacy. Within the mFARS assessment, omaveloxolone improved each component (bulbar, upper limb coordination, lower limb coordination, and upright stability) relative to placebo, although the greatest effects were on the upright stability which predicts important clinical milestones in FA (e.g, loss of ambulation).) | Low/very low | **Innovation not assigned** |
| Belzutifan | Welireg | [Welireg, INN - belzutifan](https://www.ema.europa.eu/en/documents/assessment-report/welireg-epar-public-assessment-report_en.pdf) | Conditional approval | Welireg is indicated as monotherapy for the treatment of adult patients with advanced clear cell renal cell carcinoma that progressed following two or more lines of therapy that included a PD-(L)1 inhibitor and at least two VEGF-targeted therapies.. **** (presente altra indicazione terapeutica)** | A | B | B (4 months longer survival compared to everolimus (22 months vs 18 months) | **A-B-B = moderate** | Moderate (ICIs have become standard treatment options for RCC and have greatly improved disease outcome. Initially, anti-PD-1/L1 therapies were approved as subsequent lines of care after treatment with a VEGF-targeting agent. Anti-PD-1/L1 therapies are now approved in the adjuvant setting (as monotherapy) and in the 1L setting in combination with VEGF-targeting agents or with anti-CTLA-4 checkpoint inhibitors. Although significant advances have changed the treatment paradigm for patients with advanced RCC, there continues to be a need for evidence-based options for the optimal management of pre-treated patients, particularly after immunotherapy and anti-angiogenic therapies. | Minor (The pivotal Study MK-6482-005 is an open-label, randomized phase 3 study to compare the efficacy and safety of belzutifan with that of everolimus in patients with unresectable, locally advanced or metastatic ccRCC that has progressed after prior PD-1/L1 and VEGF-targeted therapies (up to 3 prior treatment regimens). The dual primary endpoints were PFS per RECIST 1.1 as assessed by BICR and OS. Both are supported, with the knowledge that OS data are crucial for the B/R assessment. The efficacy of belzutifan in the subgroup of patients who progressed following two or more prior lines of therapy including a PD-1/L1 inhibitor and at least two VEGF-targeted therapies showed a positive effect on PFS, a numerically higher ORR and a similar OS to everolimus, authorized in this disease setting and recommended by current clinical guidelines.) | Moderate | **Innovation not assigned** |
| Repotrectinib | Augtyro | [AUGTYRO, INN - repotrectinib](https://www.ema.europa.eu/en/documents/assessment-report/augtyro-epar-public-assessment-report_en.pdf) | Conditional approval | Augtyro as monotherapy is indicated for the treatment of adult patients with ROS1-positive advanced non-small cell lung cancer (NSCLC).. **** (presente altra indicazione terapeutica)** | A | B (indicato per pazienti ROS1 positivi, che sono una piccola percentuale dei pz con NSCLC) | C (Limited evidence of a major benefit. Further studies are needed) | **A-B-C = modest** | Moderate (ROS1 rearranged NSCLC has been described as a distinct molecular type in approximately 1–2% of patients with NSCLC. Similar to ALK rearranged tumours, patients with ROS1 positive NSCLC are more commonly of younger age, have a history of never or light smoking, and have adenocarcinoma histology8. The prevalence of ROS1 positive NSCLC is higher in an Asian population of adenocarcinomas than in a Caucasian population. According ESMO clinical practice guidelines for treatment of metastatic NSCLC, a ROS1-targeted TKI is the preferred 1st line treatment in this population. Furthermore, pemetrexed-based chemotherapy is still standard of care in 2nd line after ROS1 inhibitor. Several studies have found that ROS1-positive NSCLC patients appeared to be sensitive to pemetrexed-based treatment. Crizotinib, a first generation ALK-TKI, was granted an extension of indication for ROS1 positive NSCLC in 2016. Entrectinib, a multikinase inhibitor, targeting TRK ALK and ROS1 inhibitor, was granted a full marketing authorisation in ROS1 positive NSCLC not previously treated with ROS1 inhibitor in 2020. Repotrectinib is an oral, next-generation, ATP-competitive small-molecule inhibitor of the tyrosine kinases ROS1) | Minor (Available efficacy data in terms of ORR and DoR are expected to translate into clinical benefit for the treatment of adults with ROS1 positive NSCLC, both in TKI-naïve and TKI-pretreated patients. In the TKI-naïve population, the response rate seems in line with what has been demonstrated in already approved products for the same indication although no direct comparison is available. The magnitude of this effect is such that it is expected to result in clinically relevant effects) | Moderate | **Innovation not assigned** |
| Efanesoctocog alfa | Altuvoct | [H-5968-AR-en_revised](https://www.ema.europa.eu/en/documents/assessment-report/altuvoct-epar-public-assessment-report_en.pdf) | Orphan drug | Treatment and prophylaxis of bleeding in patients with haemophilia A (congenital factor VIII deficiency). Altuvoct can be used for all age groups. | A | C2 (Medicines with FVIII activity are already available) | C (proven efficacy - long-acting action) | **A-C2-C = pharmacological, or non-innovation** | Minor (Haemophilia A is a rare X chromosome-linked bleeding disorder that occurs predominantly in males and is characterised by deficiency of functional coagulation FVIII leading to potentially life-threatening bleeding in response to trauma and recurrent bleeding into soft tissue and joints. The worldwide prevalence of haemophilia A, based on pooling across all available national registries, has been estimated to be 17.1 cases per 100 000 males. Per the current recommendations of the WFH Guidelines for the Management of Haemophilia as well as of many national and international haemophilia organisations, the standard of care for all patients with severe haemophilia A is primary prophylaxis, defined as regular therapy with FVIII replacement products or other haemostasis products to prevent spontaneous bleeding. Patients suffering from severe haemophilia A usually receive prophylactic treatment with FVIII products, which requires regular infusion every 2 to 3 days. Another option for bleeding prophylaxis is the subcutaneous administration of Hemlibra, which needs to be given only once a week to once every four weeks. Efanesoctocog alfa (BIVV001) is a recombinant fusion protein consisting of a single chain B domain- deleted human FVIII covalently linked to the D’D3 domain of human VWF via the Fc domain of human immunoglobulin G1 (IgG1) and 2 XTEN polypeptides. The prolonged half-life of Altuvoct allows an extended dosing interval of 7 days, which will to some extent decrease the burden of the regular prophylactic infusion for patients with severe haemophilia A, compared to the currently available extended half-life products.) | Important ( The presented clinical data provide sufficient evidence that a once weekly administration of BIVV001 is effective for prophylaxis, treatment of bleeding episodes and perioperative management in subjects with severe haemophilia A. No dose adjustment has been performed across all age groups of the entire Phase 3 study programme. Even in the paediatric study no dose adjustment has been done despite shortening of the weekly dosing interval would have been an option. Number of breakthrough bleedings was low in all age groups. Overall, in the pivotal study EFC16293 and in the paediatric study EFC16295, clinical efficacy of Altuvoct has been demonstrated for prophylactic treatment using 50 IU/kg weekly and on-demand treatment of 50 IU/kg as single dose in an adequate number of adults, adolescents and children with severe haemophilia A | Moderate | **Innovation not assigned** |
| Mirvetuximab soravtansine | Elahere | [ELAHERE, INN-Mirvetuximab soravtansine](https://www.ema.europa.eu/en/documents/assessment-report/elahere-epar-public-assessment-report_en.pdf) | Orphan drug | as monotherapy is indicated for the treatment of adult patients with folate receptor-alpha (FRα) positive, platinum-resistant high grade serous epithelial ovarian, fallopian tube, or primary peritoneal cancer who have received one to three prior systemic treatment regimens. | A | B (in patients less or non-responsive to standard treatment) | A (increase in PFS and OS, although of short duration) | **A-B-A = important** | Moderate (According to current ESMO guidelines, epithelial ovarian cancer (EOC) represents a heterogeneous spectrum of disease entities at a clinical, pathological and molecular level. Ovarian cancer is the second most lethal gynaecological malignancy worldwide behind cervical cancer and the first in developed countries. Maintenance therapy with poly adenosine diphosphate-ribose polymerase (PARP) inhibitors has been incorporated into first-line treatment for EOC, with multiple randomized studies showing a benefit in progression-free survival (PFS) over placebo (SOLO1 [Moore et al. 2018], ATHENA-mono [Monk et al. 2022], PRIMA [González-Martín et al. 2019]). For patients with homologous recombination deficiency, including BRCA mutation, outcomes have improved considerably with the use of PARP inhibitors as first-line maintenance therapy. Despite the incorporation of maintenance therapy into the treatment paradigm, most patients with advanced EOC relapse during or after treatment with platinum-containing regimens) | Moderate (The MIRASOL study met its primary endpoint PFSINV, however the PFS time gain was only 1.6 months with MIRV compared with ICC (5.62 vs. 3.98 months). The key secondary endpoint OS was also met: the median OS with MIRV was 16.46 (14.46, 24.57) months and 12.75 (10.91, 14.36) months with ICC, meaning a 3.71 month OS benefit in favor of MIRV. These results are clinically relevant, as advanced platinum-resistant ovarian cancer is a lethal disease, in which currently available chemotherapies offer limited efficacy. To conclude, the efficacy of MIRV as monotherapy, despite the uncertainties and deficiencies highlighted in the discussion, is considered established for the treatment of adult patients with folate receptor-alpha (FRα) positive, platinum-resistant high grade serous epithelial ovarian, fallopian tube, or primary peritoneal cancer who have received one to three prior systemic treatment regimens.) | Moderate | **Innovation assigned** |
| Exagamglogene autotemcel | Casgevy | [Casgevy; INN-exagamglogene autotemcel](https://www.ema.europa.eu/en/documents/assessment-report/casgevy-epar-public-assessment-report_en.pdf) | Orphan drug, PRIME - priority medicine, Advanced Therapy, Conditional approval | Casgevy is indicated for the treatment of transfusion dependent β thalassemia (TDT) in patients 12 years of age and older for whom haematopoietic stem cell (HSC) transplantation is appropriate and a human leukocyte antigen (HLA) matched related HSC donor is not available.. **** (presente altra indicazione terapeutica)** | A | B (Regular transfusions or the drugs used are not consistently effective, are not risk-free (hydroxyurea) and compromise the quality of life; bone marrow transplants are not always feasible due to the lack of a donor) | C (limited evidence of major benefits, preliminary results, requiring confirmation) | **A-B-C=C innovazione modesta** | Moderate (Transfusion-dependent β-thalassemia (TDT), a rare disease, is the most severe form of β-thalassemia, which is characterized by severe anaemia requiring regular lifetime transfusions of RBCs, which lead to a number of serious complications, including multi-organ damage. The recommended treatment for TDT is lifelong regular blood transfusions, usually administered every two to five weeks, to maintain the pre-transfusion haemoglobin level 9.5-10.5 g/dl. Currently, the only curative treatment options for TDT are allogeneic haematopoietic stem cell transplant (allo-HSCT) and a lentiviral gene therapy (betibeglogene autotemcel; Zynteglo), whose EU marketing authorization had been withdrawn upon request of the marketing authorization holder.) | Minor (Casgevy (exagamglogene autotemcel or exa-cel) is an autologous CD34⁺ hHSPCs modified by CRISPR/Cas9-mediated gene therapy medicinal product with editing of the erythroid enhancer region of the BCL11A gene. Clinical data is being generated in the ongoing TDT interventional study 111 and the respective long-term follow-up study 131. Patients were recruited from EU and USA. The patients were TDT patients, 12 to 35 years of age, eligible for HSCT, but lacking an available suitable donor. Relevant endpoints are the primary endpoint TI12 (defined as maintaining weighted average Hb ≥9 g/dL without RBC transfusions for at least 12 consecutive months any time after exa-cel infusion.) and the secondary endpoint “RBC transfusion free”. In study 111, 39 of 42 (92.9%) subjects in the Primary Efficacy Set (PES) achieved TI12. All subjects in the PES who met the primary endpoint remained transfusion free for the duration of follow-up; the mean (SD) transfusion free duration while maintaining weighted Hb ≥9 g/dL was 23.6 (7.8) months, starting 60 days after the last RBC transfusion. While the early efficacy results are promising, for a comprehensive data set especially longer FU is needed to demonstrate that the benefit of the treatment is maintained in the long term. This is reflected by considering a conditional instead of a full approval, with generation of respective long-term follow-up data post-authorisation as part of specific obligations.) | Low/very low | **Innovation not assigned** |
| Polihexanide | Akantior | [AKANTIOR - EPAR](https://www.ema.europa.eu/en/documents/assessment-report/akantior-epar-public-assessment-report_en.pdf) | Orphan drug | is indicated for the treatment of Acanthamoeba keratitis in adults and children from 12 years of age. | A | A (the active substance itself is an antimicrobial and antiparasitic present in other medicines in Germany, therefore it has not been declared a new active substance, however the "novelty" lies in the creation of the eye drops) | C (limited evidence of a major benefit, Study design and results are not very conclusive) | **A-A-C = moderate** | Important (Acanthamoeba keratitis is a rare, painful, and sight-threatening infection caused by Acanthamoeba species, a family of free-living, ubiquitous protozoa commonly found in water, dust, and soil. This infection results from invasion of ocular tissue through a corneal lesion. The incidence of Acanthamoeba keratitis is described to be between 1 and 4 per million (Nielsen et al 2019, Randag et al 2019). There are currently no drugs licensed for use in Acanthamoeba keratitis (AK) and no standard treatment is established across the EU. Thus, patients and ophthalmologists use unlicensed or off-label treatment options. Diamidines and biguanides are considered effective cysticidal anti-amoebic agents. The current approach for the treatment for Acanthamoeba keratitis includes biguanides (e.g. polihexanide or chlorhexidine) with or without the addition of diamidines (e.g. propamidine or hexamidine). According to literature, in most cases additional concomitant therapy is applied (i.e. especially antibiotics, antifungals, corticosteroids and pain killers). In cases of medical failure, a surgical intervention (as amniotic membrane transplantation, deep anterior lamellar keratoplasty or penetrating keratoplasty) may be required in approximately one third of patients) | Absent (Polihexanide 0.8 mg/ml is intended to be applied as eye drops for the treatment of Acanthamoeba keratitis (AK). The product was granted an orphan designation in 2007 (EU/3/07/498). No treatment is licensed for the treatment of Acanthamoeba keratitis in the EEA. Two prospectively planned clinical studies were conducted, study 042/SI (042) for dose selection and study 043/SI (043) to assess efficacy and safety compared to an unlicensed active comparator chosen to be a combination of polihexanide 0.2 mg/ml and propamidine 1 mg/ml in AK patients; the latter is the single pivotal trial supporting this application. Study 043 was initially planned to demonstrate superiority of the monotherapy treatment (polihexanide 0.8mg/ml + placebo; in this report sometimes referred to just polihexanide 0.8mg/ml) over the unlicensed combination therapy (polihexanide 0.2 mg/ml and propamidine 1 mg/ml), but included the option to switch to non-inferiority as per guideline. In total, 69 and 66 subjects were assigned to treatment with 0.8 mg/ml polihexanide + placebo and 0.2 mg/ml polihexanide + 1 mg/ml propamidine, respectively. All except one subject received at least one dose of study drug, resulting in 134 subjects in the Safety Analysis Set. Regarding the primary outcome of trial 043, the 12-month clinical resolution rate is slightly lower for polihexanide +placebo (84.8% for FAS and 87.1% for the PPAS) compared to polihexanide +propamidine (88.5% for FAS and 88.5% for the PPAS). Thus, superiority was not demonstrated, as the difference is not statistically significant (neither at 5% nor at 10% significance level in either analysis set). The difference in proportion of clinical resolution rate is -0.04 with 90% CI (-0.14, 0.07) for the FAS and -0.02 with 90% CI (-0.13, 0.08) for the PPAS. The lower limit of the 90% CI is above the pre-defined non-inferiority margin of 20% in both analysis sets, i.e. polihexanide +placebo is not more than 20% worse than polihexanide +propamidine.) | Low/very low | **Innovation not assigned** |
| Pegzilarginase | Loargys | [Loargys, INN-pegzilarginase;](https://www.ema.europa.eu/en/documents/assessment-report/loargys-epar-public-assessment-report_en.pdf) | Exceptional circumstances, Orphan drug | is indicated for the treatment of arginase 1 deficiency (ARG1 D), also known as hyperargininemia, in adults, adolescents and children aged 2 years and older. | A | A | B (the therapeutic effect studied was only the arginine levels and not the overall picture of the disease; the long-term effects of the drug were not studied, especially as regards the maintenance of therapeutic efficacy) | **A-A-B = important** | Moderate (ARG1 D (arginase-1 deficiency) is a rare, debilitating, progressive, inherited, neurodegenerative, metabolic disease associated with increased arginine and its toxic metabolites. ARG1-D is associated with significant reductions in quality of life, increased morbidity (lower-limb spasticity), and premature mortality. It is an autosomal recessive disease caused by a deficiency in the ARG1 enzyme, which is an essential step in the urea cycle. Current management approaches for ARG1-D mainly consist of dietary protein restriction to reduce arginine, together with essential amino acid (EAA) supplementation. International guidelines for ARG1- D focus on the reduction of plasma arginine to levels of. Other treatments, such as ammonia scavengers, help to control excessive ammonia levels. In addition, spasmolytic agents and anti-epileptics are applied as symptomatic treatments. Still, ammonia scavengers only target a part of the symptoms, i.e. those related to (acute) hyperammonaemia. However, they do not reduce the formation of the toxic metabolite of arginine, which causes the neurotoxicity that is the hallmark of the disease (spasm and motor disorders). Liver transplantation has been reported to normalise arginine and ammonia levels and ameliorate neurological symptoms in individual cases, and significantly improve neurodevelopmental and growth delay. At the same time, liver transplantation is available to only a small fraction of patients and carries a significant risk of morbidity and mortality, also related to life-long required use of immunosuppressant agents.) | Moderate (Pegzilarginase is a modified, cobalt-substituted, PEGylated recombinant human ARG1 enzyme with enhanced stability, more potent catalytic activity, and an extended half-life compared to the native enzyme. The main study (300A) is a randomised, placebo-controlled trial, on top of previously used and unchanged standard of care with a protein-restricted diet (plus nitrogen scavengers if applicable). The choice of a placebo control is considered adequate, as no pharmacological treatments are available for this disease. Pegzilarginase was administered in weekly IV infusions. The dose was adjusted based on the individual PD response of the arginine levels. ARG1-D was defined as having one of the following signs: elevated plasma arginine levels, a mutation analysis that resulted in a pathogenic variant, or reduced red blood cell arginase activity. Only a single randomised trial is performed (300A), which may be understood given the ultra-rarity of the disorder. The double-blind placebo-controlled study phase of the main study was 24 weeks. After this, patients were eligible for the LTE phase, where all patients could receive pegzilarginase. The LTE study phase is still on-going and the final study report will be provided in the post-marketing setting. Change in plasma arginine was selected as the primary endpoint. It has been confirmed that pegzilarginase could significantly reduce arginine levels in ARG1-D patients who had hyperarginaemia, despite a protein-restricted diet. Pegzilarginase demonstrated a 76.7% (95% CI 67.1, 83.5%) reduction in GLS mean ratio for change of plasma arginine at Week 24 from baseline relative to placebo. Illustrative of the magnitude of the primary outcomes is that the responder rates of subjects achieving arginine levels below the treatment goal of 200 μM, or 115uM, was 90.5% of subjects in the pegzilarginase group versus 0 in the placebo group. However, the two clinical key secondary outcomes failed to meet their endpoints at a statistically significant level.) | Low/very low | **Innovation assigned** |
| Odronextamab | Ordspono | [Ordspono, INN-odronextamab](https://www.ema.europa.eu/en/documents/assessment-report/ordspono-epar-public-assessment-report_en.pdf) | Orphan drug, Conditional approval | as monotherapy is indicated for the treatment of adult patients with relapsed or refractory follicular lymphoma (r/r FL) after two or more lines of systemic therapy. **** (presente altra indicazione terapeutica)** | A | B (indicated in patients who have failed first and second line treatment) | C [Clinically meaningful rates of ORR/CR, however, the CHMP requires the completion of clinical studies to confirm approval) | **A-B-C = innovazione modesta** | Moderate (FL is the most common indolent lymphoma in the US and Europe, accounting for approximately 25% of all B-NHLs. The median age at diagnosis of FL is 65 years. Autoimmune disease and some occupational exposures have been identified as risk factors of FL. Observation (watch-and-wait) is the standard practice for asymptomatic patients with low tumour burden FL. In the frontline setting guidelines recommend that treatment is only initiated upon occurrence of symptoms, or continuous progression with threatened organ function, or one or more of the modified GELF criteria (including, but not restricted to, bulky disease and cytopenias). When systemic treatment is indicated, immunochemotherapy with an anti-CD20 monoclonal antibody and chemotherapy, or monotherapy with an anti-CD20 monoclonal antibody, is the first-line standard of care. Patients who progress after 1 line of therapy may be considered for other standard regimens used in frontline treatment based on their performance status. The combination of (i) lenalidomide with rituximab, and (ii) obinutuzumab with bendamustine are the only available therapies that have full approvals in the EU in second line for patients with r/r FL. In the third line setting, available treatment options include certain treatment options approved for first- and/or second-line FL. Options for these patients also include CAR-T products (axicabtagene ciloleucel, tisagenlecleucel), CD20xCD3 bispecific antibody (mosunetuzumab), PI3K inhibitors, BTK inhibitor, and radioimmunotherapy.) | Minor (Clinically meaningful rates of ORR/CR, and clinically meaningful durations of responses have been shown in both DLBCL as well as FL. Despite the single-arm study design, exploratory nature of the pivotal trial, and limited sample size, the high activity of odronextamab is evident in the agreed indications. Nevertheless, the CHMP considers the following measures necessary to address the missing efficacy data in the context of a conditional MA: • In order to provide further evidence of efficacy and safety of odronextamab in relapsed or refractory follicular lymphoma, the MAH will provide results from study R1979-ONC-22102, a phase 3, open label, randomised trial to compare the efficacy and safety of odronextamab in combination with lenalidomide versus rituximab in combination with lenalidomide in relapsed or refractory participants with follicular lymphoma and marginal zone lymphoma.) | Low/very low | **Innovation not assigned** |
| Epcoritamab | Tepkinly | [Tepkinly; INN-epcoritamab](https://www.ema.europa.eu/en/documents/assessment-report/tepkinly-epar-public-assessment-report_en.pdf) | Conditional approval | as monotherapy is indicated for the treatment of adult patients with relapsed or refractory diffuse large B-cell lymphoma (DLBCL) after two or more lines of systemic therapy.. **** (presente altra indicazione terapeutica)** | A | B (indicated in patients who have failed first and second line treatment) | C (limitations of the ongoing clinical trial were found: Therefore, the results should be interpreted with caution and confirmation of efficacy in the R/R DLBCL population is required for a full approval) | **A-B-C = modest** | Minor (The standard first-line therapy for LBCL and DLBCL as per the recommendations of NCCN and ESMO is R-CHOP (or dose-adjusted R-EPOCH). Substituting vincristine for Polivy leads to a superior PFS in patients with an intermediate- or high-risk prognosis by IPI (Tilly 2021). Although approximately two thirds of patients survive for 5 years after first line treatment, up to 50% of the patients become refractory to treatment or relapse (Crump et al., 2017). For patients with relapsed or refractory (r/r) DLBCL, standard therapy currently entails salvage chemotherapy with non-cross resistant chemotherapy in second line, followed by autologous hematopoietic stem cell transplantation (ASCT) in medically fit patients (Philip et al., 1995). For DLBCL patients after two or more lines of systemic therapy several therapies are available: Chemo-immunotherapy, CAR-T, Zynlonta is an ADC targeting CD19 authorised by the EC in December 2022, Pixantrone is a cytotoxic aza-anthracenedione approved for multiply relapsed or refractry aNHL (3rd and 4th line of treatment). | Minor (Clinically relevant responses and duration of response were observed in the study population of LBCL subtypes DLBCL NOS, HGBL, PMBCL as well as in FL3B who are R/R after two or more lines of systemic therapy. The single arm trial phase 1/2 design introduces inherent limitations as the observed therapeutic effect might be subject to various sources of bias. In addition, efficacy may be overestimated in such a study design. Therefore, the results should be interpreted with caution and confirmation of efficacy in the R/R DLBCL population is required for a full approval. The CHMP considers the following measures necessary to address the missing efficacy data in the context of a conditional MA: • In order to confirm the safety and efficacy of epcoritamab in the treatment of R/R DLBCL after two or more lines of systemic therapy, the results of the primary (including final OS analysis) and final safety and efficacy analyses for study GCT3013-05 should be submitted. • In order to confirm the safety and efficacy of epcoritamab in the treatment of R/R DLBCL after two or more lines of systemic therapy, the final CSR for study GCT3013-01 should be submitted.) | Moderate | **Innovatività non riconosciuta** |
| rADAMTS13 | Adzynma | [Adzynma; INN-rADAMTS13](https://www.ema.europa.eu/en/documents/assessment-report/adzynma-epar-public-assessment-report_en.pdf) | Exceptional circumstances, Orphan drug | Treatment of congenital thrombotic thrombocytopenic purpura (cTTP) due to ADAMTS13 deficiency | A | B (the transfusion approach is considered) | C (Taking into account the rarity of the disease and the low incidence of TTP events during the clinical trial period, no inferential statistical analyses but presentation of data via descriptive statistics only were planned and presented for the primary endpoint. As no firm conclusions regarding the efficacy of the product to prevent the acute attacks can be drawn, the totality of the data has to be taken into account in order to support a positive benefit-risk evaluation, and uncertainties remain with regard to efficacy, safety and dosing, which cannot realistically be addressed via longer follow-up or another clinical trial in the post marketing setting.) | **A-B-C = modest** | Moderate (Congenital thrombotic thrombocytopenic purpura is an ultra-rare, life-threatening, chronic, and debilitating blood clotting disorder with an estimated prevalence of 0.5 to 4 cases/million. There are no medications approved for routine prophylactic treatment of cTTP. Current SoC treatment centers around the principle of replacing the missing ADAMTS13 enzyme through on-demand or regular prophylactic infusions of available plasma-based therapies, mostly FFP or S/D plasma. The therapeutic rationale for ADAMTS13 replacement therapy is well understood in this genetic enzyme deficiency disorder and it has been demonstrated that plasma replacement therapy may reduce the frequency and severity of cTTP episodes and has been shown to resolve 96% of acute TTP events in patients with cTTP. TAK-755 (previously known as “BAX 930” or “SHP655”) is recombinant ADAMTS13.) | Absent (Phase 3 trial 281102 is a randomised, controlled, open-label, 2 period crossover study with a single arm continuation phase evaluating the safety and efficacy of TAK-755 in the prophylactic and on demand setting. Subjects with severe congenital thrombotic thrombocytopenic purpura (ADAMTS13 activity via the prophylactic cohort and be randomised to 6 months of treatment either with standard of care or TAK-755 followed by 6 months of treatment with the other treatment modality and subsequently enter the continuation phase of another 6 months of TAK-755 treatment. In the case of an acute TTP event, subjects could enter the trial via the on-demand cohort for a randomised treatment of the acute event with either SoC or TAK-755. Subjects could then proceed to the prophylactic cohort, re-enroll for another acute event or leave the trial. The primary efficacy outcome was the incidence of acute TTP events among subjects receiving either TAK-755 or SoC prophylactically during the corresponding treatment periods. Phase 3b trial TAK-755-3002 is a single arm, open label continuation study investigating the efficacy and safety of prophylactic and on demand treatment with rADAMTS13 treatment up to 3 years. In total approximately 48 subjects (36 adults and 12 adolescent or paediatric subjects) were to be enrolled in the prophylactic cohort. Due to the very low number of acute TTP events, no inferential statistical analysis but only descriptive statistics are presented for the primary endpoint, i.e. the incidence of acute TTP events among subjects receiving either TAK-755 or SoC prophylactically during the corresponding treatment periods. At the end, the outcome of 0 events can either be seen as a direct consequence of satisfactory efficacy of ERT using rDAMTS13, or as a result of the unpredictable natural disease course of hereditary TTP featuring discontinuous event incidence, or (most likely) a mixture of both. Thus, no firm conclusions regarding the efficacy of TAK-755 in the prophylactic setting can be drawn from the primary outcome of the pivotal study.) | Low/very low | **Innovation not assigned** |
| Tofersen | Qalsody | [Qalsody, INN-tofersen](https://www.ema.europa.eu/en/documents/assessment-report/qalsody-epar-public-assessment-report_en.pdf) | Exceptional circumstances, Orphan drug | is indicated for the treatment of adults with amyotrophic lateral sclerosis (ALS), associated with a mutation in the superoxide dismutase 1 (SOD1) gene. | A | B (a drug with these characteristics (mutation of the SOD1 gene) has no equal alternatives; in more general terms a treatment (of poor efficacy) for ALS is represented by risuzole, and was represented by edaravone (Radicava) withdrawn in 2019) | C (Significant clinical results are lacking; further studies are required. Although the effects of tofersen on biomarkers have been demonstrated, there is no evidence that these effects correspond to real clinical benefits.) | **A-B-C = innovazione modesta.** | Maximum (ALS is a rare disease. The global prevalence of ALS is estimated to be approximately 4.42 per 100,000. Riluzole, the most widely approved ALS treatment, received its first global approval for treatment of ALS in 1995 (US) based on 2 studies that demonstrated that the time to tracheostomy or death was approximately 2 months longer in participants receiving riluzole than in those receiving placebo. Edaravone received its first approval for the treatment of ALS in 2015 in Japan based on a single study in Japanese patients with ALS of Grade 1 or 2 in the Japan ALS Severity Classification. In Europe, a MA application was filed (EMEA/H/C/004938) and later withdrawn in 2019. Edaravone (Radicava) is not approved in the EU. Edaravone is available through a compassionate use programme in some EU countries. | Minore (Despite its clear biological activity, tofersen did not significantly improve function in patients with SOD1- ALS in the 28-week single pivotal trial in comparison to placebo. This may be explained by a lag-time between effects on biomarkers and clinical effects, which appears biologically plausible and is supported by anecdotal evidence from two other ASOs used in patients with neurodegenerative diseases, including another form of ALS. Indeed, longer-term results showed consistent favourable effects on functional, strength, QoL and survival-related endpoints for early start vs. delayed start tofersen treatment, irrespective of the analyses performed and over long periods of time, at least up to week 104. In addition, the reported survival times in treated patients clearly exceed those expected from natural history data. Although, efficacy of tofersen in SOD1 ALS has not been proven in a confirmatory sense all results are consistent with a reasonably likely disease-modifying treatment effect. | Low/very low | **Innovation not assigned** |
| Talquetamab | Talvey | [Talvey; INN-talquetamab](https://www.ema.europa.eu/en/documents/assessment-report/talvey-epar-public-assessment-report_en.pdf) | Orphan drug, PRIME - priority medicine, Conditional approval | is indicated as monotherapy for the treatment of adult patients with relapsed and refractory multiple myeloma, who have received at least 3 prior therapies, including an immunomodulatory agent, a proteasome inhibitor, and an anti CD38 antibody and have demonstrated disease progression on the last therapy. | A | B (in patients progressing even after three previous therapies) | C (preliminary data, conditional approval, efficacy to be confirmed) | **A-B-C = modest** | Important (The treatment landscape for relapsed or refractory multiple myeloma (RRMM) has changed in recent years. Current treatment of MM includes glucocorticoids, chemotherapy, primarily alkylating agents, high dose chemotherapy followed by ASCT, proteasome inhibitors (PIs, such as bortezomib, carfilzomib and ixazomib), immunomodulatory agents (such as thalidomide, lenalidomide and pomalidomide), monoclonal antibodies ((mAbs), such as daratumumab, isatuximab and elotuzumab) and the histone deacetylase inhibitor panobinostat. Common standard regimens include either a PI or an IMiD in combination with dexamethasone with or without a monoclonal antibody such as daratumumab. The triplet combination of bortezomib, lenalidomide, and dexamethasone (VRd) is a standard. Newer classes of medications including XPO1 inhibitors (selinexor) and antibody drug conjugates targeting BCMA (belantamab mafodotin-blmf) and also CAR T cell products (Idecaptagene vicleucel, Cilacaptagene autoleucel) have been approved by EMA. The latest was talquetamab, which is a bsAb that targets GPRC5D and CD3, humanised IgG4 bispecific antibody designed to target the CD3 receptor complex on T cells and on GPRC5D-expressing multiple myeloma cells, resulting in T cell activation and  subsequent lysis of GPRC5D-expressing multiple myeloma cells.) | Minor (Subjects in Part 3 (Phase 2) Cohorts A and C were requested to have previously received ≥3 prior lines of therapy (including at least one PI, one IMiD, and an anti-CD38 monoclonal antibody) with no previous exposure to T cell redirection therapies such as CAR-T or bispecific antibodies. The available data from pivotal study MMY-1001 showed that treatment with talquetamab, in a heavily pre-treated population of patients with RR MM, resulted in high ORRs with a relevant proportion of deeper responses, in line with what observed in similar settings with other T-cell redirection therapies. Study 64407564MMY1001 met its pre specified primary endpoint (30% for Cohorts A and C and 15% for cohort B) demonstrating a clinically significant improvement in Overall Response Rate (ORR). Overall, despite the significant uncertainties related to the limited sample size, the available data in subjects who had received prior anti-BCMA CAR T cell therapies and bispecific monoclonal antibodies suggested that deep and durable responses can still be obtained with talquetamab. Study 64407564MMY3002 is a multicentre, randomised, open-label, Phase 3 study to determine whether talquetamab in combination with daratumumab and pomalidomide (Tal- DP; Arm A) and Assessment report EMA/365426/2023 Page 101/155 talquetamab in combination with daratumumab (Tal-D; Arm C) have better efficacy respectively than the combination of daratumumab, pomalidomide and dexamethasone (DPd; Arm B) in participants with relapsed or refractory multiple myeloma who have previously received at least 1 prior line of therapy. Approximately 810 patients will be randomised in a 1.1.1 ratio to Tal-DP, Tal-D and DPd.) | Low/very low | **Innovation not assigned** |
| Adagrasib | Krazati | [Krazati, INN-adagrasib](https://www.ema.europa.eu/en/documents/assessment-report/krazati-epar-public-assessment-report_en.pdf) | Conditional approval | as monotherapy is indicated for the treatment of adult patients with advanced non-small cell lung cancer (NSCLC) with KRAS G12C mutation and disease progression after at least one prior systemic therapy. | A | B | C (evidence for efficacy of Krazati in the treatment of adult patients with advanced NSCLC with KRAS G12C mutation who have received at least one prior systemic therapy is limited and comes from an uncontrolled single-arm trial (849-001, KRYSTAL-1) | **A-B-C= modest** | Moderate (In January 2022, the European Commission granted a Conditional Marketing Authorisation (CMA) to Lumykras (sotorasib) for the treatment of patients with previously treated NSCLC harbouring the KRAS G12C mutation. Such approval was based on pharmacological, efficacy and safety data from the CodeBreak 100 study, which showed favourable results from sotorasib in the overall population, with an ORR of 37.1% (95% CI: 28.6, 46.2) and a median DOR of 11.1 months (95% CI: 6.9, 15.0). Interim results from the confirmatory trial for such CMA (CodeBreak 200) are already available and indicate a median PFS of 5.6 months [95% CI 4.3–7.8] for sotorasib vs 4.5 months [3.0–5.7] for docetaxel; hazard ratio 0.66 [0.51–0.86]; p=0.0017 (de Langen, 2023). Overall survival is confounded due to cross-over. | Minor (The main efficacy dataset to support this application is constituted by primary analysis results of Cohort A (n=116) from the phase 2 segment of KRYSTAL-1 (Study 849-001), a phase 1/2, open-label, multi-cohort, single-arm trial conducted in the US. ORR, as assessed by BICR, is the primary endpoint of efficacy to support this application, whereas DOR, PFS and OS are secondary endpoints. The primary efficacy dataset of Cohort A from Study KRYSTAL-1 is constituted by the 116 patients that were treated with adagrasib. At initial data cut-off (DCO) 15-JUN-2021, 48 patients out of 116 were considered confirmed responders by retrospective BICR, accounting for an ORR of 41.4% (95% 32.3, 50.9). The inferior limit of the 95% CI excludes the 23% specified benchmark used in sample size calculations, rendering the primary analysis successful. At the updated DCO, no new objective responses were reported between the two data cutoffs, and ORR by BICR remained at 41.4%. Evidence for efficacy of adagrasib in the targeted population is limited and comes from an uncontrolled single-arm trial (849-001, KRYSTAL-1). Evidence for efficacy of Krazati in the treatment of adult patients with advanced NSCLC with KRAS G12C mutation who have received at least one prior systemic therapy is limited and comes from an uncontrolled single-arm trial (849-001, KRYSTAL-1). At the latest data cut-off (DCO) of 15 October 2021, 48 out of 116 patients treated were considered confirmed responders by retrospective BICR, leading to an ORR of 41.4% (95% 32.3; 50.9). The median duration of response (mDOR) was estimated at 8.5 months (95% 6.2; 13.8). The long-term benefit of Krazati is unclear since its impact on time-to-event endpoints, i.e., PFS and OS, cannot be reliably estimated in the context of an uncontrolled trial) | Low/very low | **Innovation not assigned** |
| Retifanlimab | Zynyz | [Zynyz, INN-retifanlimab](https://www.ema.europa.eu/en/documents/assessment-report/zynyz-epar-public-assessment-report_en.pdf) | Orphan drug | is indicated as monotherapy for the first-line treatment of adult patients with metastatic or recurrent locally advanced Merkel cell carcinoma (MCC) not amenable to curative surgery or radiation therapy. | A | C2 (The drug avelumab already exists with the same indication) | C (according to the CHMP encouraging results, promising results, but a study with a control group is missing. Furthermore, the lack of comparison with avelumab is not justified) | **A-C2-C = pharmacological, or non-innovation** | Moderate (Merkel cell carcinoma is an aggressive, life-threatening, cutaneous malignancy with a poor outcome when advanced. The annual incidence of MCC worldwide is ranging between 0.10 and 1.6 per 100,000 persons. Surgery and/or radiation therapy are indicated and, potentially, curative for local-regional disease. . Bavencio (avelumab) has been granted marketing authorisation by the European Commission for the treatment of MCC. Anti-PD-(L)1 therapies are currently recommended as first-line treatment for locally advanced/metastatic MCC. Retifanlimab is an immunoglobulin G4 (IgG4) monoclonal antibody that binds to the programmed death receptor-1 (PD-1) and blocks its interaction with its ligands PD-L1 and PD-L2.) | Minor (The pivotal study supporting this marketing authorisation application (MAA) is INCMGA 0012-201 (POD1UM-201), an ongoing, open-label, single-arm, multiregional, Phase 2 study in chemotherapy-naïve patients with advanced Merkel cell carcinoma (MCC). Objective response rate as primary endpoint is appropriate for single-arm trials. Anti-tumour activity observed in the trial can directly be linked to the activity of the study drug and, thereby, interpretable without a control arm. It should, however, be noted that (complete) spontaneous regression has been reported, occasionally. Sample size of approximately 60 patients gives a 80% power to exclude the lower 95% CI of 30% was made based on the avelumab approved in MCC, which showed a confirmed ORR of 39.7% with lower 95% CI Assessment report EMA/122634/2024 Page 128/191 of 30.7% in chemotherapy-naïve patients. Retifanlimab shows promising anti-tumour activity in patients with advanced/metastatic MCC, as evident by the primary and (key) secondary outcomes. The ORR was high (52.3% [95% CI: 39.5, 64.9]) and median DOR not reached (61.8% of the responses were ≥ 12 months). The primary objective of the study was met (i.e., the lower limit of the 95% CI exceeded 30%). PFS and OS results are difficult to interpret without a control arm. Nevertheless, PFS results are encouraging, with median PFS 12.7 months (95% CI: 7.3, 24.9). Retifanlimab shows promising antitumour activity that likely translates into meaningful benefit. The treatment effects observed in the pivotal trial are consistent with those estimated for other anti-PD-(L)1 therapies in MCC. The ORR was high (53.5% [95% CI: 43.3, 63.5]) and response was durable (25.3 months [95% CI: 14.2, NE]), and PFS results are encouraging.) | Low/very low | **Innovation not assigned** |
